# Nucleosides Rescue Replication-Mediated Genome Instability of Human Pluripotent Stem Cells

**DOI:** 10.1016/j.stemcr.2020.04.004

**Published:** 2020-05-14

**Authors:** Jason A. Halliwell, Thomas J.R. Frith, Owen Laing, Christopher J. Price, Oliver J. Bower, Dylan Stavish, Paul J. Gokhale, Zoe Hewitt, Sherif F. El-Khamisy, Ivana Barbaric, Peter W. Andrews

**Affiliations:** 1 Department of Biomedical Science, University of Sheffield, Western Bank, Sheffield S10 2TN, UK; 2 Krebs Institute, Department of Molecular Biology and Biotechnology, University of Sheffield, Sheffield S10 2TN, UK

**Keywords:** pluripotent, DNA, damage, replication, stress, nucleosides, mitotic, errors, human

## Abstract

Human pluripotent stem cells (PSCs) are subject to the appearance of recurrent genetic variants on prolonged culture. We have now found that, compared with isogenic differentiated cells, PSCs exhibit evidence of considerably more DNA damage during the S phase of the cell cycle, apparently as a consequence of DNA replication stress marked by slower progression of DNA replication, activation of latent origins of replication, and collapse of replication forks. As in many cancers, which, like PSCs, exhibit a shortened G1 phase and DNA replication stress, the resulting DNA damage may underlie the higher incidence of abnormal and abortive mitoses in PSCs, resulting in chromosomal non-dysjunction or cell death. However, we have found that the extent of DNA replication stress, DNA damage, and consequent aberrant mitoses can be substantially reduced by culturing PSCs in the presence of exogenous nucleosides, resulting in improved survival, clonogenicity, and population growth.

## Highlights

• Human pluripotent stem cells are susceptible to replication stress
• Nucleosides modulate replication stress and DNA damage in human PSCs
• Nucleosides alleviate mitotic errors and improve the growth kinetics of human PSCs
• These findings provide a means to reduce the incidence of recurrent genetic change

## Teaser

Halliwell et al. document the susceptibility of human PSCs to replication stress, which may lead to genetic instability. The results presented show that this phenotype can be moderated by culture in the presence of exogenous nucleosides. The findings provide an approach to reducing the incidence of recurrent genetic changes that may compromise the use of human PSCs in regenerative medicine.

## Introduction

Human pluripotent stem cells (PSCs) often acquire genetic changes on prolonged culture, which pose concerns for their use in research and regenerative medicine (Amps et al., 2011, Taapken et al., 2011). The acquisition of these changes during culture necessarily first requires mutation and then selection of those mutations that provide a growth advantage. Whereas selection accounts for the recurrent nature of the variants commonly reported (Draper et al., 2004, Olariu et al., 2010), the mechanisms of acquired mutation in PSCs remain largely unexplored. Recent work has started to elucidate environmental conditions that have an impact on the mutational burden of PSCs, highlighting oxidative stress as a major contributor to the acquisition of single nucleotide variation in PSCs (Thompson et al., 2020). In addition to such extrinsic influences, intrinsic properties of PSCs are also thought to play a role in the maintenance of their genome stability (Ahuja et al., 2016). The defining features of PSCs that make them distinct from somatic cells are the pluripotent gene regulatory network responsible for maintaining the pluripotent state (Pera et al., 2000), and a rapid cell cycle due to an abbreviated G1 compared with somatic cells (Becker et al., 2006). In mouse embryonic stem cells (ESCs), the abbreviated G1 was shown to cause replication stress (Ahuja et al., 2016), a collective term for interruption of the DNA replication process, which manifests as a slowing or stalling of the DNA replication fork, resulting in DNA breaks upon a fork collapse (Zeman and Cimprich, 2014). Replication stress due to an abbreviated G1 is also implicated in cancer development, wherein high levels of cyclin E drive the premature entry of cells into the S phase in the absence of sufficient nucleotide pools needed for normal progression of the replication fork (Bester et al., 2011). Consequently, the nucleotide-deficient cells exhibit replication stress, predisposing them to DNA damage and subsequent genome instability (Bester et al., 2011, Burrell et al., 2013).

A high level of DNA damage in human PSCs has been reported previously (Simara et al., 2017, Vallabhaneni et al., 2018), but the underlying causes and means of alleviating the damage remain unknown. Here, we show that an increased susceptibility to DNA damage in PSCs compared with their isogenic differentiated cells is caused by persistent replication stress in PSCs. Importantly, we demonstrate that the addition of exogeneous nucleosides to the culture medium restores the replication dynamics and reduces the level of genome damage and incidence of mitotic aberrations in PSCs. Finally, we show that nucleoside supplementation also improves survival of PSCs, demonstrating that replication stress-associated genome damage is a major cause of cell death in PSC cultures.

## Results and Discussion

**Figure 1:**
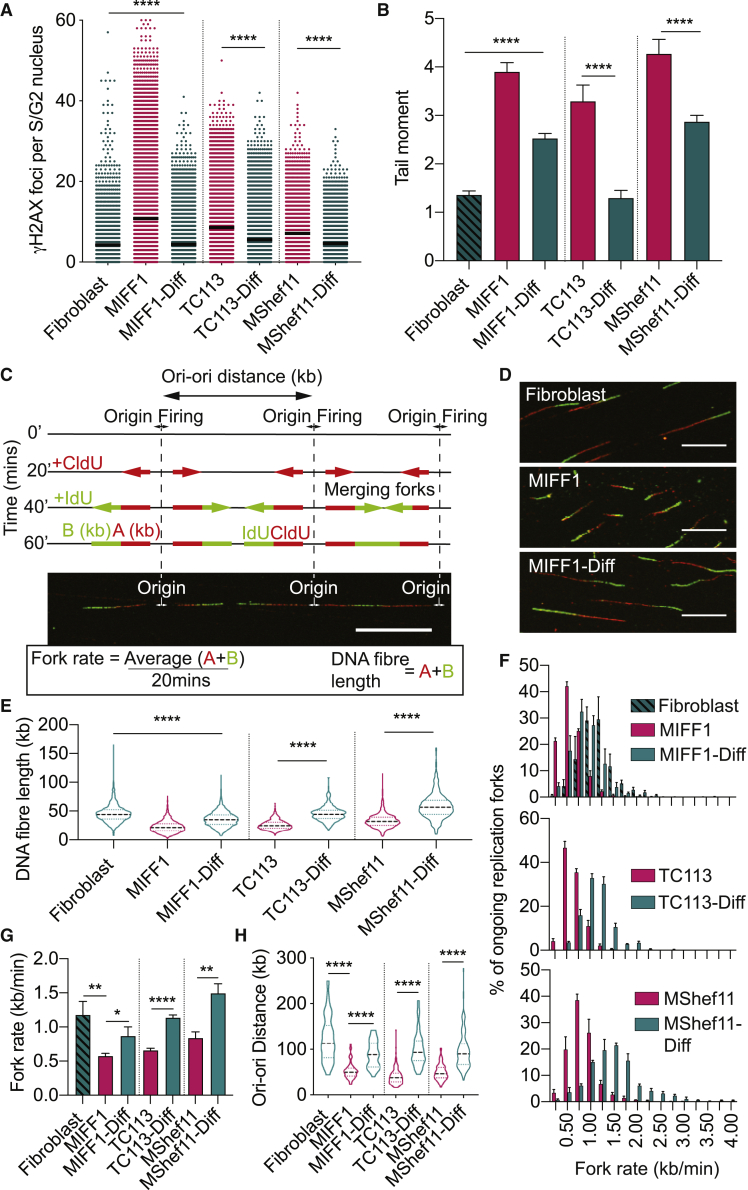
Replication Stress and a Susceptibility to DNA Damage is a Characteristic of Undifferentiated Human PSCs Data in the figure compare undifferentiated and differentiated cell states. MIFF1 was compared with its parent fibroblast line (Fibroblast) and with its differentiated derivative (MIFF1-Diff). TC113 and MShef11 were compared with their differentiated derivatives (TC113-Diff and MShef11-Diff). (A) The number of γH2AX foci per S-/G2-phase cell. The S/G2 phase was determined from nuclear DNA content. Data points in (A) represent individual cells and are the results from three independent experiments; the center line indicates the mean. Two-tailed t test, ^∗∗∗∗^p < 0.0001 (n > 100 cells per cell line per experiment). (B) Average tail moment from neutral comet assays. Data displayed are from three independent experiments ± SEM. Two-tailed t test, ^∗∗∗∗^p < 0.0001 (n ≥ 300 cells per cell line per experiment). (C) Schematic of DNA fiber analysis. Sequential 20-min pulses of CldU and IdU labeled the progressing replication forks. Measurements of CldU and IdU lengths enable the analysis of replication fork dynamics. Scale bar, 10 μm. (D) Representative DNA fibers are shown for Fibroblast, MIFF1, and MIFF1-Diff. Scale bar, 10 μm. (E) Combined length of CldU and IdU in individual fibers (*n* > 200 forks per cell line per experiment, n = 3 experiments). Median distance, 25^th^ and 75^th^ quartiles are presented. Two-tailed t test, ^∗∗∗∗^p < 0.0001. (F) Distribution of replication fork rates (n > 200 forks per cell line per experiment, n = 3 experiments). Data are mean values from each experiment ± SEM. (G) Mean fork rates from (F) ± SD. Two-tailed *t* test, ^∗^p < 0.05, ^∗∗^p < 0.01, ^∗∗∗∗^p < 0.0001 (n = 3 experiments). (H) Distribution of adjacent origins distance measurements (Ori-ori). Median distance, 25^th^ and 75^th^ quartiles are presented. Two-tailed t test, ^∗∗∗∗^p < 0.0001 (n > 30 per cell line, n = 3 experiments).

DNA double-strand breaks (DSBs) are a particularly detrimental type of DNA damage. Unrepaired, or erroneously repaired, DSBs can jeopardize genome stability by leading to mitotic aberrations and structural and chromosomal instability (Ichijima et al., 2010, Janssen et al., 2011), like those frequently observed in human PSCs (Amps et al., 2011). The human induced PSC line MIFF1 (Desmarais et al., 2016) exhibited an increased number of γH2AX foci (Figure 1A), known to mark sites of DSBs, compared with the fibroblast from which it was reprogrammed and to its differentiated derivatives, obtained by treatment with CHIR99021 for 5 days (Figure S1). Two other PSC lines that we examined, TC113 (Baghbaderani et al., 2015) and MShef11 (Thompson et al., 2020), showed a similarly increased level of DNA damage compared with their differentiated derivatives (Figures 1A and S1). These observations were confirmed by a neutral comet assay that showed an increased tail moment in the undifferentiated cells in each case (Figure 1B). We also compared the growth of MIFF1 in four commercially available feeder-free media and found similar levels of γH2AX foci, although slightly lower in cells grown in Nutristem (Figure S2A). Overall, the results demonstrate that a high level of genome damage is associated with the pluripotent state and decreases upon differentiation.

A common cause of DSBs during S phase in cancer cells is the slowing, stalling, and collapse of replication forks, recognized as DNA replication stress (Ichijima et al., 2010). To analyze the replication dynamics in undifferentiated and differentiated cells, we utilized the DNA fiber assay. Here, the newly synthesized DNA is pulse labeled successively with thymidine analogs, cholorodeoxyuridine (CldU) and iododeoxyuridine (IdU), for 20 min each and then visualized by fluorescently labeled antibodies (Figure 1C). By measuring the total length of the CldU and IdU labeling in each fiber, we found a decrease in the length of newly synthesized fibers in the undifferentiated state (Figures 1C–1E). Replication fork speed, calculated by measuring the average length of labeled fibers, was significantly slower in undifferentiated PSCs compared with their isogenic somatic counterparts (Figures 1C, 1F, and 1G). Further, we found an increase in the abundance of origins of DNA replication as demonstrated by a decrease in replication origin-to-origin distance (Ori-ori) in PSCs (Figures 1C and 1H). Overall, these results show that DNA replication in pluripotent cells is considerably perturbed, predisposing them to DNA damage, notably DSBs.

**Figure 2:**
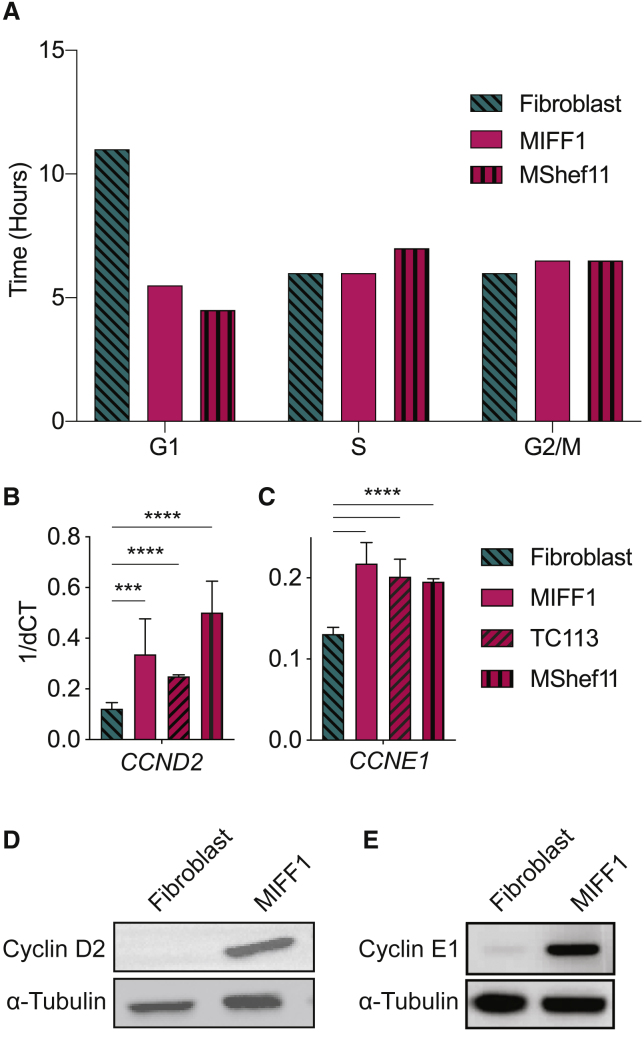
Cell-Cycle Dynamics and Cyclin Expression in Human PSCs Are Candidates for Replication Stress Initiation (A) Cell-cycle phase time determined from time-lapse microscopy (Fibroblast, n = 44; MIFF1, n = 76; MShef11, n = 31) and EdU pulse-chase analysis of asynchronous cells (minimum of 10,000 events recorded during fluorescence-activated cell sorting analysis). (B and C) RT-qPCR gene expression data from MIFF1, TC113, and MShef11 for *CCND2* (B), *CCNE* (C). Data in (B) and (C) are means ± SD. Two-tailed t test, ^∗∗∗^p < 0.001, ^∗∗∗∗^p < 0.0001 (n = 3 experiments). (D and E) Representative western blot of protein expression for cyclin D2 (D), cyclin E1 (E).

The association of genome damage with the pluripotent rather than the somatic state of the same cell line, suggests that features linked to pluripotency impart replication stress on PSCs. A pertinent key difference between the pluripotent and somatic cell state is the rapid progression of PSCs through G1, driven by atypical expression of cyclins (Becker et al., 2006, Ahuja et al., 2016). By time-lapse microscopy and 5-ethynyl-2′-deoxyuridine (EdU) pulse-chase analysis, we found that the human PSC line, MIFF1, exhibited a reduced cell-cycle time when compared with its parent fibroblast line (Figure 2A). Specifically, the abbreviated cell-cycle time was solely due to a truncated G1 (Figure 2A). Consistent with the reduction in the length of G1, cyclin D2 (*CCND2*) and cyclin E (*CCNE1*), which are known to drive the Rb-E2F pathway and allow rapid progression of cells through G1 (Hinds et al., 1992, Lundberg and Weinberg, 1998), were highly expressed in the undifferentiated MIFF1 compared with the corresponding parent fibroblast line (Figures 2B–2E). We confirmed these findings in TC113 and MShef11, which also exhibited a similar short G1 and enhanced expression of cyclin D2 and E (Figures 2A–2C).

**Figure 3:**
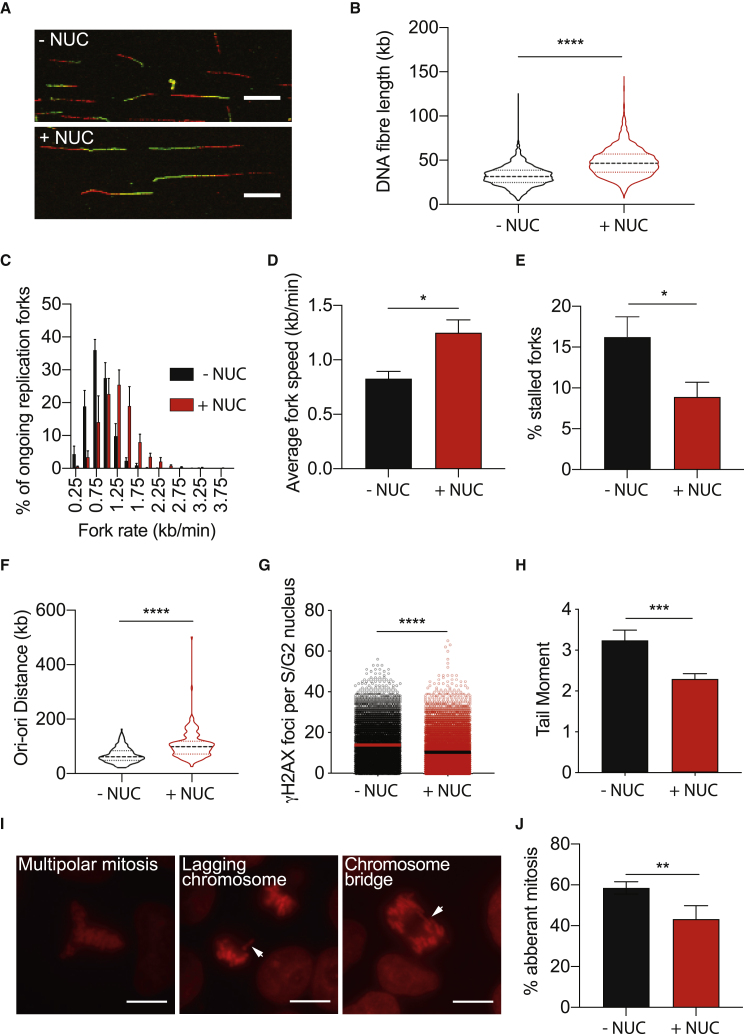
Exogenously Supplied Nucleosides Alleviate Replication Stress in Human PSCs (A) Representative images of DNA fibers in the absence of nucleosides (−NUC) and in the presence of exogenous nucleoside (+NUC) conditions. Scale bar, 10 μm. (B) Combined length of CldU and IdU in individual fibers (n > 200 forks per cell line per experiment, n = 3 experiments). Median distance, 25^th^ and 75^th^ quartiles are presented. Two-tailed t test, ^∗∗∗^p < 0.001. (C) Distribution of replication fork rates (n > 200 forks per condition per experiment, n = 3 experiments). Data are means ± SEM. (D) Average fork rates from (C). Data are means ± SD. Two-tailed t test, ^∗^p < 0.05 (n = 3 experiments). (E) Frequency of CldU-only tracts that denote a stalled replication fork (n > 700 forks per condition per experiment, n = 3 experiments). Data are means ± SD. Two-tailed t test, ^∗^p < 0.05. (F) Distribution of adjacent origins distance measurements (Ori-ori). Median distance, 25^th^ and 75^th^ quartiles are presented. Two-tailed t test, ^∗∗∗∗^p < 0.0001 (n > 150 per cell line, n = 3 experiments). (G) γH2AX foci per S-/G2-phase cell (determined from DNA content). Each data point is the measurement of an individual cell; the center line indicates the mean. Two-tailed t test, ^∗∗∗∗^p < 0.0001 (n > 100 cells per condition per experiment, n = 3 experiments). (H) Average tail moment from neutral comet assay experiments. Data are means ± SD. Two-tailed t test, ^∗∗∗^p < 0.001 (n ≥ 300 cells per condition per experiment, n = 3 experiments). (I and J) Mitotic errors observed from fluorescently labeled chromatin (histone H2B-RFP). Representative images of mitotic errors (I). White arrows point to mitotic error in each case. Scale bar, 10 μm. Average frequency of mitotic errors observed (J). Data are means ± SD. Unpaired t test, ^∗∗^p < 0.01 (n = 13–43 mitosis assessed per condition per experiment, n = 4 experiments).

We reasoned that the short G1 may have an impact on genome damage of PSCs, since overexpression of cyclin D2 and E in cancer cells has also been reported to enforce an abbreviation of G1 and consequent replication stress, which can be modulated by exogenous nucleosides (Bester et al., 2011, Takano et al., 2000). We tested whether the addition of exogenous nucleosides would improve the replication dynamics of human PSCs. After an initial titration of nucleosides, using γH2AX as a readout of genome damage, we chose a formulation 15μM cytidine, 15μM guanosine, 15μM uridine, 15μM adenosine, and 6μM thymidine. The addition of these exogenous nucleosides increased DNA fiber lengths and replication fork speed in MIFF1 to levels comparable with those observed in its differentiated derivatives (Figures 3A–3D compared with Figures 1D–1G). In addition, we noted fewer CldU-only tracts, indicating fewer forks stalled prior to the addition of the second thymidine analog, IdU (Figure 3E). There was also a decrease in replication origin density, with Ori-ori distances in MIFF1 now comparable with those observed in the parent fibroblast line and following the differentiation of MIFF1 (Figure 3F compared with Figure 1H), suggesting that, as a consequence of slower fork speed, the cells were firing from dormant origins in the absence of exogenous nucleosides. We confirmed these findings in TC113 and MShef11, which show a similar increase in replication fork speed and Ori-ori distance with the addition of exogenous nucleosides (Figures S2B–S2F). Under these conditions we observed a marked decrease in the frequency of DSBs in MIFF1, TC113, and MShef11 as indicated by a reduction in the number of γH2AX foci per S-phase and G2-phase cells upon addition of nucleosides (Figures 3G and S2G) and a decrease in tail moment measured using the neutral comet assay (Figure 3H). Similarly, exogenous nucleosides also reduced the number of γH2AX foci when the cells were cultured in alternative human PSC culture media, Nutristem and E8 (Figure S2H), while also retaining the ability to differentiate (Figure S3). Overall, these results indicate that susceptibility to DNA damage observed in the undifferentiated PSCs is a consequence of replication stress that can be alleviated by exogenous nucleosides.

A detrimental consequence of replication stress is the presence of under-replicated regions that can persist into mitosis and hinder chromosome separation (Bester et al., 2011, Burrell et al., 2013, Ichijima et al., 2010). This in turn can lead to mitotic aberrations, including chromosome bridges, lagging chromosomes, and the formation of micronuclei (Crasta et al., 2012, Ichijima et al., 2010, Janssen et al., 2011). Using time-lapse microscopy of MIFF1 cells stably transfected with H2B-RFP to fluorescently label chromatin, we tracked the progression of cells through mitosis. Consistent with previous reports (Lamm et al., 2016, Zhang et al., 2019), we observed a high incidence of mitotic errors in human PSCs. However, the incidence of these errors was significantly decreased in cells cultured in the presence of nucleosides (Figures 3I and 3J), indicating that replication stress in human PSCs is a cause of mitotic errors.

**Figure 4:**
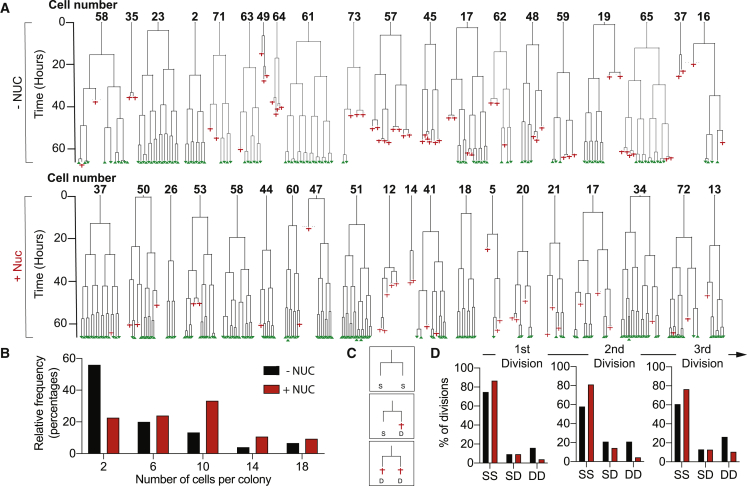
Exogenous Nucleosides Improve Survival and the Ability of Human PSCs to Re-enter the Cell Cycle Post Plating (A) Time-lapse data tracing the growth of individual MIFF1 cells that reach the first cell division plotted as lineage trees. A random sample of 20 cells (full set of 75 cells shown in Figure S4) grown without exogenous nucleosides (top, −NUC) and in the presence of exogenous nucleosides (bottom, +NUC). Where the trees fork indicates a cell division, red crosses show cell death, and surviving cells at the end of the time lapse are noted with a green triangle. Time is displayed on the y axis. (B–D) Summary data of the lineage tree analysis (B). Histogram of the distribution colony sizes at the end of the time-lapse experiment (C). Schematic illustrating the scoring method used in (D). After each division, the fate of the daughter cells was recorded: both daughter cells surviving (SS), one daughter cell survives and one dies (SD), or both daughter cells die (DD). (D) Individual bar charts show the frequency of SS, SD and DD daughter cell fates following the first, second and third divisions (left to right) after plating (D). Cell culture media without exogenous nucleosides (black) and grown in the presence of exogenous nucleosides (red) conditions are shown.

To investigate the consequences of these observations for the proliferation of human PSCs, we used time-lapse microscopy to track the growth of single cells through successive divisions. When MIFF1 cells were seeded at low density, 68% of those that attached went on to divide in normal culture medium, whereas 79% entered mitosis in medium supplemented with nucleosides. This is consistent with our previous observation that human PSCs activate apoptosis in response to replication stress (Desmarais et al., 2012, Desmarais et al., 2016). Of those that did enter mitosis, 59% went on to form colonies of two or more cells under standard culture conditions, with many cells dying after the first and subsequent divisions (Figures 4A and S4A). By contrast, fewer cells died following division when exogenous nucleosides were added and 91% of cells went on to form colonies (Figures 4A and S4B) with substantially greater final size (Figure 4B). In the absence of added nucleosides, there was a consistently higher number of abortive cell divisions involving the death of both daughter cells (Figures 4C and 4D), a result that would be anticipated if the mitotic errors caused by DNA replication stress are catastrophic for both daughter cells.

Taken together, our results demonstrate that human PSCs, compared with somatic cells, are predisposed to high levels of replication stress, manifest by slower rates of DNA synthesis, activation of latent origins of replication, and the stalling of replication forks. One consequence is their susceptibility to double-stranded DNA breaks that, in turn, may lead to genomic rearrangements during mitosis (Ichijima et al., 2010, Janssen et al., 2011). These results reflect the requirement for rapid replication of human PSCs enabled by a truncated G1 (Becker et al., 2006, Becker et al., 2010) that impairs the preparation of these cells for the ensuing DNA replication. However, a further feature of human PSCs is that, unlike somatic cells, they tend to undergo apoptosis in response to replication stress, so minimizing the appearance of mutant cells. This might reflect the demands of cell proliferation in the early embryo in which any genomic damage in even one cell could be catastrophic for the whole embryo (Desmarais et al., 2012, Desmarais et al., 2016). Indeed, in a separate study, we have found that the overall mutation rate in human PSCs is rather low (Thompson et al., 2020), despite their propensity to DNA damage. Nevertheless, that human PSCs tend to accumulate particular recurrent mutations and genomic rearrangements, most likely reflects selection for growth advantages among those few variants that escape apoptosis. It is notable that resistance to apoptosis is a common feature of many recurrent variants that do arise in human PSCs (Avery et al., 2013, Barbaric et al., 2014, Merkle et al., 2017). Our observation that exogenous nucleosides substantially reduced DNA replication stress in human PSCs, perhaps compensating for metabolic changes that stem from their shortened G1 and relaxed G1/S transition, provides a means to reduce the incidence of recurrent genetic changes that may compromise the use of human PSCs for disease modeling and regenerative medicine.

## Experimental Procedures

### Human Pluripotent Stem Cell Culture

Two human iPSC lines, MIFF1 and TC113, and one human ESC line, MShef11, were used in this study. MIFF1 had been reprogrammed at the University of Sheffield, Centre for Stem Cell Biology, from a human foreskin fibroblast, CCD-112Sk (ATCC, CRL-2429) using a vector-free mRNA reprogramming system (Desmarais et al., 2016) (hPSCreg: https://hpscreg.eu/cell-line/UOSi001-A). The second human iPSC line, TC113, was acquired from RUCDR Infinite Biologics. It had been reprogrammed from CD34+ umbilical cord blood cells using a non-integrating episomal vector reprogramming system (Baghbaderani et al., 2015) (hPSCreg: https://hpscreg.eu/cell-line/RUCDRi002-A). Finally, the human ESC line, MShef11, had been isolated at the University of Sheffield, Centre for Stem Cell Biology, under good manufacturing practice-like conditions in a cleanroom setting (HFEA license R115-8-A (Centre 0191) and HTA license 22510). The fresh embryo was a surplus/unsuitable embryo for *in vitro* fertilization treatment, donated from the assisted conception unit and cultured to the blastocyst stage using *in vitro* fertilization medium (Medicult) before explantation onto human feeders (Thompson et al., 2020) (hPSCreg: https://hpscreg.eu/cell-line/UOSe015-A).

These PSCs were all cultured on vitronectin (VTN-N) recombinant human protein (Thermo Fisher Scientific, A14700). Culture vessels were coated with 200μL/cm^2^ of vitronectin that had been diluted to 6μg/mL with PBS and incubated at 37°C for at least 1 h. The cells were maintained in feeder-free conditions, batch fed daily with Essential 8 (Chen et al., 2011), mTeSR1 (STEMCELL Technologies, 85850), NutriStem XF (Biological Industries, 05-100-1A) or Essential 8 medium (Chen et al., 2011) without fibroblast growth factor 2 (FGF2) but supplemented with StemBeads FGF2 (800μL per 100 mL) (StemCultures, SB500), and incubated in a 37°C, 5% CO_2_ humidified incubator. Passaging of cells was performed by clump dissociation using ReLeSR (STEMCELL Technologies, 05,873) following the manufacturer’s guidelines.

### Differentiation of Human PSCs

Human PSCs were grown for 5 days in E8 medium (Chen et al., 2011)without FGF2 and transforming growth factor β but supplemented with 10 μM CHIR99021 (Tocris, 4423). Loss of pluripotency was confirmed by an RT-qPCR panel of self-renewal, mesoderm, endoderm, and ectoderm genes (Table S1) and by immunofluorescence staining and imaging of NANOG.

### Fibroblast Cell Culture

Fibroblasts (ATCC, CRL2429) were grown in Iscove's modified Dulbecco's medium (Thermo Fisher Scientific, 12440053) with 20% fetal bovine serum (HyCLone, SV30160.03). Cells were passaged using TrypLE cell dissociation enzyme (Thermo Fisher Scientific, 12504013) following the manufacturer's guidelines. Cells were maintained at 37°C and 5% CO_2_ in a humidified incubator.

### Nucleoside Supplementation

EmbryoMax Nucleosides 100X (Merck, ES-008-D) were added to mTeSR, E8, Nutristem, or StemBeads cell culture media at a final concentration of 0.5× (15μM cytidine, 15μM guanosine, 15μM uridine, 15μM adenosine, and 6μM thymidine). All experiments were performed after 72 h in culture with the supplementation of nucleosides.

## Author Contributions

P.W.A. and I.B. oversaw the project. J.A.H., I.B., and P.W.A devised the experiments. J.A.H. performed most cell biology experiments with help from T.J.R.F., O.L., C.J.P., D.S., and O.J.B. J.A.H. and T.J.R.F. performed the differentiations of the human PSCs. J.A.H. and O.L. performed embryoid body formation and pluripotency-associated antigen expression analysis. J.A.H., C.J.P., D.S., and O.J.B. performed time-lapse lineage tree experimentation and analysis. Z.H. oversaw the derivation of the hiPSC2 cell line. P.W.A., I.B., P.J.G., and S.F.E. provided experimental advice. The manuscript was drafted by J.A.H., P.W.A., and I.B.

## Supplemental Information

Document S1. Supplemental Experimental Procedures and Figures S1–S4

Document S2. Article plus Supplemental Information
