# Supplementary material for: Nucleosides Rescue Replication-Mediated Genome Instability of Human Pluripotent Stem Cells
Source: Stem Cell Reports. 2020 May 14;14(6):1009–17. doi: 10.1016/j.stemcr.2020.04.004 (PMC7355123; doi:10.1016/j.stemcr.2020.04.004)
Supplement: Document S1. Supplemental Experimental Procedures and Figures S1–S4 [file mmc1.pdf]

Stem Cell Reports, Volume *14*

Supplemental Information

Nucleosides Rescue Replication-Mediated Genome Instability of Human Pluripotent Stem Cells

Jason A. Halliwell, Thomas J.R. Frith, Owen Laing, Christopher J. Price, Oliver J. Bower, Dylan Stavish, Paul J. Gokhale, Zoe Hewitt, Sherif F. El-Khamisy, Ivana Barbaric, and Peter W. Andrews

Supplemental Figures

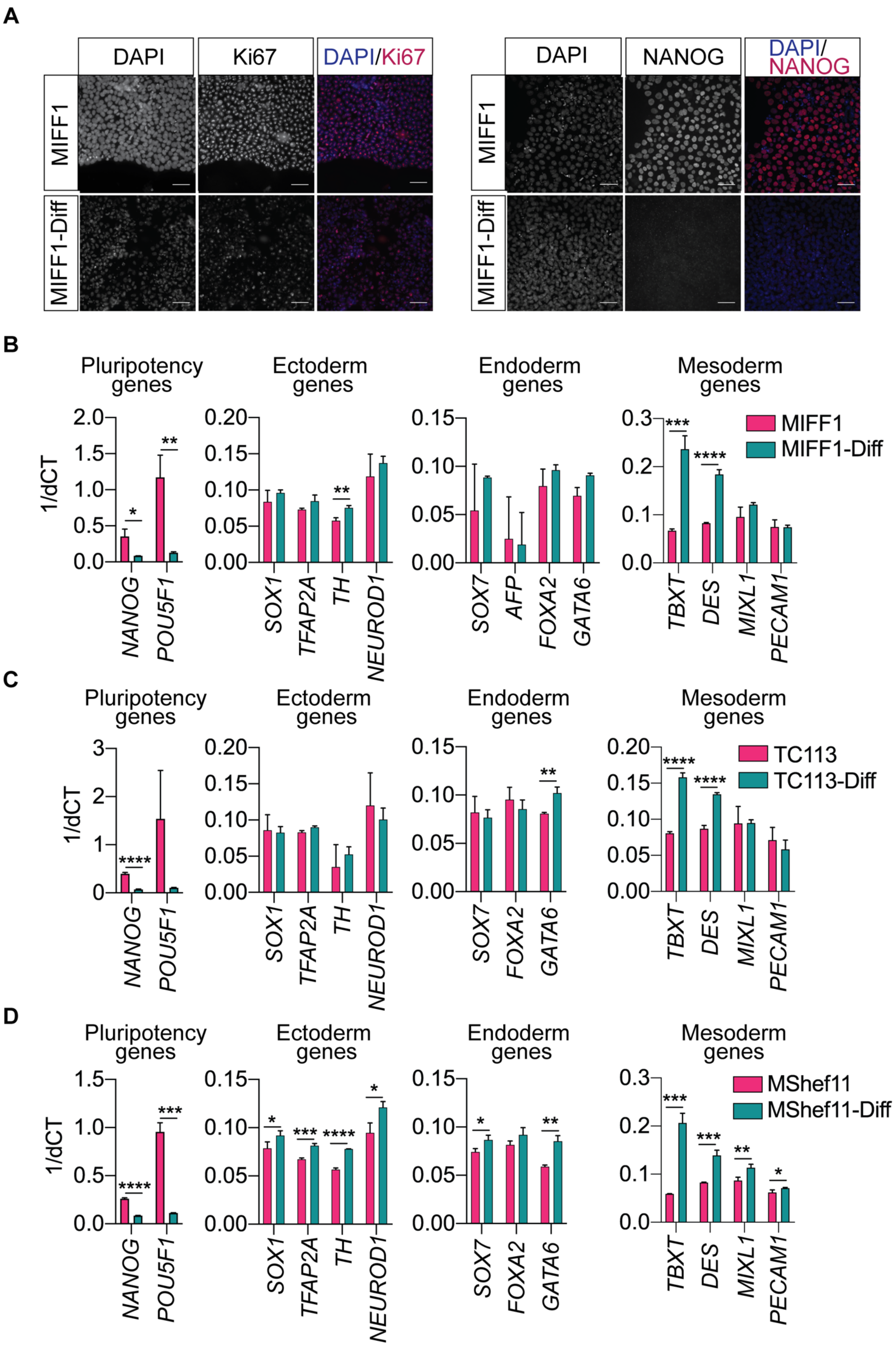

Figure S1| Differentiation of human PSC lines to obtain isogenic differentiated derivatives. Figure S1 supports figure 1 by providing details of the human PSC differentiation A, Representative immunofluorescence images of MIFF1 and MIFF1 Differentiated cells (MIFF1-Diff) stained for Ki67 and NANOG, with nuclei counterstained with Hoechst 33342. Scale bar, 50µm. **B-D,** RT-qPCR gene expression data of MIFF1 **(B),** TC113 **(C)** and MShef11 **(D)** compared to their differentiated derivatives. Genes associated with pluripotency, ectoderm, endoderm and mesoderm are displayed (left to right). Data in **B**-**D** are mean ± s.d., two-tailed *t*-test, *P<0.05, **P<0.01, ***P<0.001, ****P<0.0001, (*n* = 3 experiments).

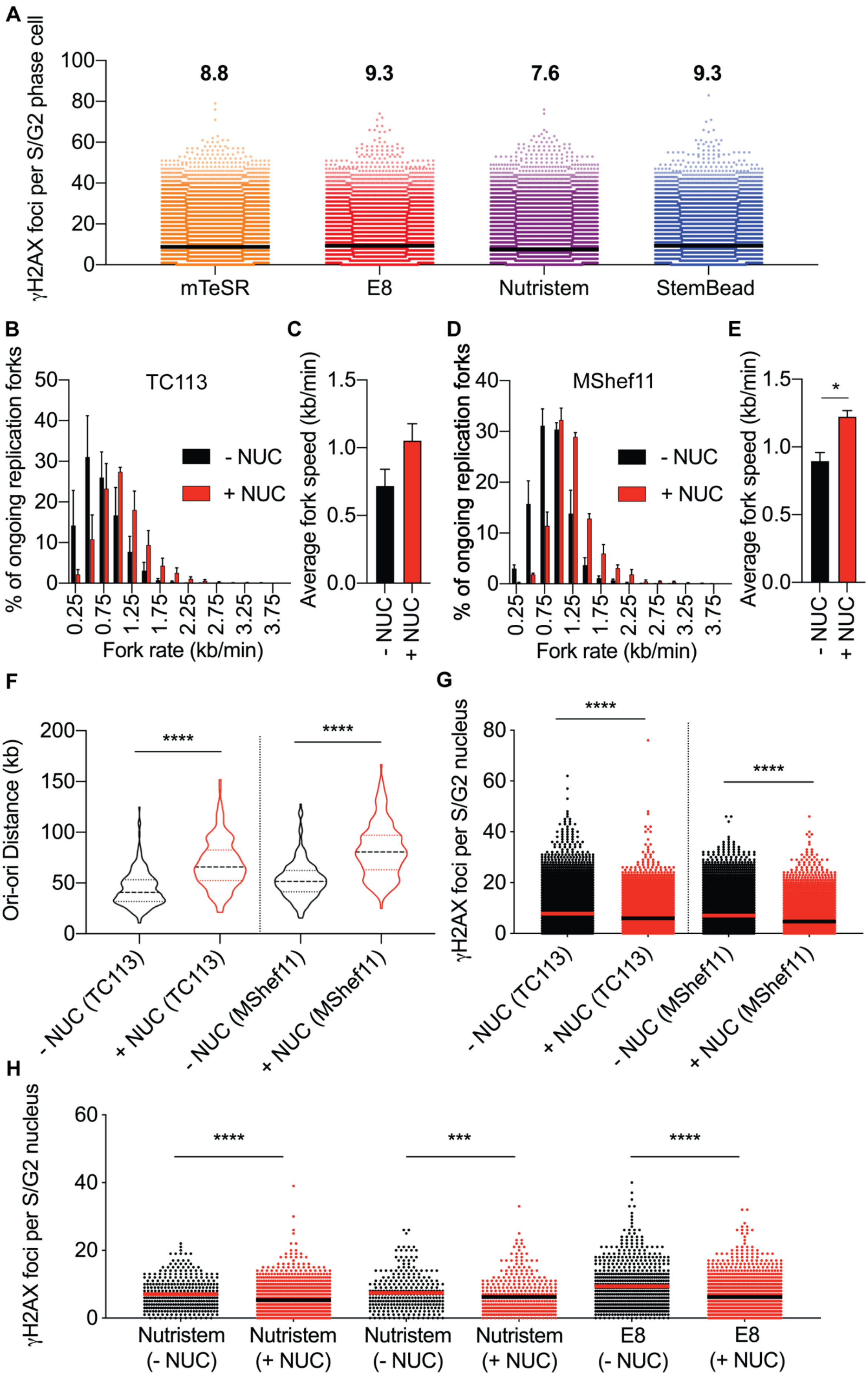
A
γH2AX foci per S/G2 phase cell
8.8
9.3
7.6
9.3
mTeSR
E8
Nutristem
StemBead
B
TC113
% of ongoing replication forks
- NUC
+ NUC
Fork rate (kb/min)
C
Average fork speed (kb/min)
- NUC
+ NUC
D
MShef11
% of ongoing replication forks
- NUC
+ NUC
Fork rate (kb/min)
E
Average fork speed (kb/min)
*
- NUC
+ NUC
F
Ori-ori Distance (kb)
****
****
- NUC (TC113)
+ NUC (TC113)
- NUC (MShef11)
+ NUC (MShef11)
G
γH2AX foci per S/G2 nucleus
****
****
- NUC (TC113)
+ NUC (TC113)
- NUC (MShef11)
+ NUC (MShef11)
H
γH2AX foci per S/G2 nucleus
****
***
****
Nutristem (- NUC)
Nutristem (+ NUC)
Nutristem (- NUC)
Nutristem (+ NUC)
E8 (- NUC)
E8 (+ NUC)

Figure S2 | Replication stress and a susceptibility to DNA damage is a characteristic of undifferentiated human PSC, Figure S2 additional data in support of figure 3. A, The number of γH2AX foci per S/G2 phase cell when MIFF1 was grown in mTeSR, E8, Nutristem or StemBeads. The S/G2 phase was determined from nuclear DNA content. Data points represent individual MIFF1 cells and are the results from three independent experiments, centre line indicates the mean, the mean numerical value is also presented above each plot. The mean numbers of γH2AX foci was similar for all media, except that it was slightly lower in the case of Nutristem, two-tailed *t*-test, ****P<0.0001. **B-F,** Additional data from DNA fibre assays performed on TC113 **(B,C)** and MShef11 **(D,E)** in absence of exogenous nucleosides (- NUC) or in the presence of exogenous nucleoside (+ NUC)**. B,D** Distribution of replication fork rates (*n* > 200 forks per cell line per experiment, *n* = 3 experiments), data presented is the mean value from each experiment ± s.e.m. **C,E** Mean fork rates. Data is mean ± s.d., two-tailed *t*-test, *P<0.05**.** (*n* > 200 forks per cell line per experiment, *n* = 3 experiments) **F,** Distribution of adjacent origins distance measurements (Ori-ori). Median distance, 25th and 75th quartiles are presented, two-tailed *t*-test, ****P<0.0001 (*n* > 160 per cell line, *n* = 3 experiments). **G,** Number of γH2AX foci per S/G2 phase cell (determined from DNA content). Comparison made between TC113 and MShef11 cells grown without nucleosides (- NUC) and with nucleoside (+ NUC). Data in **G** are the results from 3 independent experiments, centre line is the mean. two-tailed *t*-test, ****P<0.0001 (*n* > 100 cells per cell line per experiment). **H,** The number of γH2AX foci per S/G2 phase MIFF1 cell when grown in Nutristem or E8 without the addition of nucleosides (- NUC) and when these media were supplemented with exogenous nucleosides (+ NUC). Each plot shows an individual experiment, with each point representing the value from a single MIFF1 cell. Centre line indicates the mean. two-tailed *t*-test, ***P<0.001, ****P<0.0001 (*n* > 100 cells per experiment).

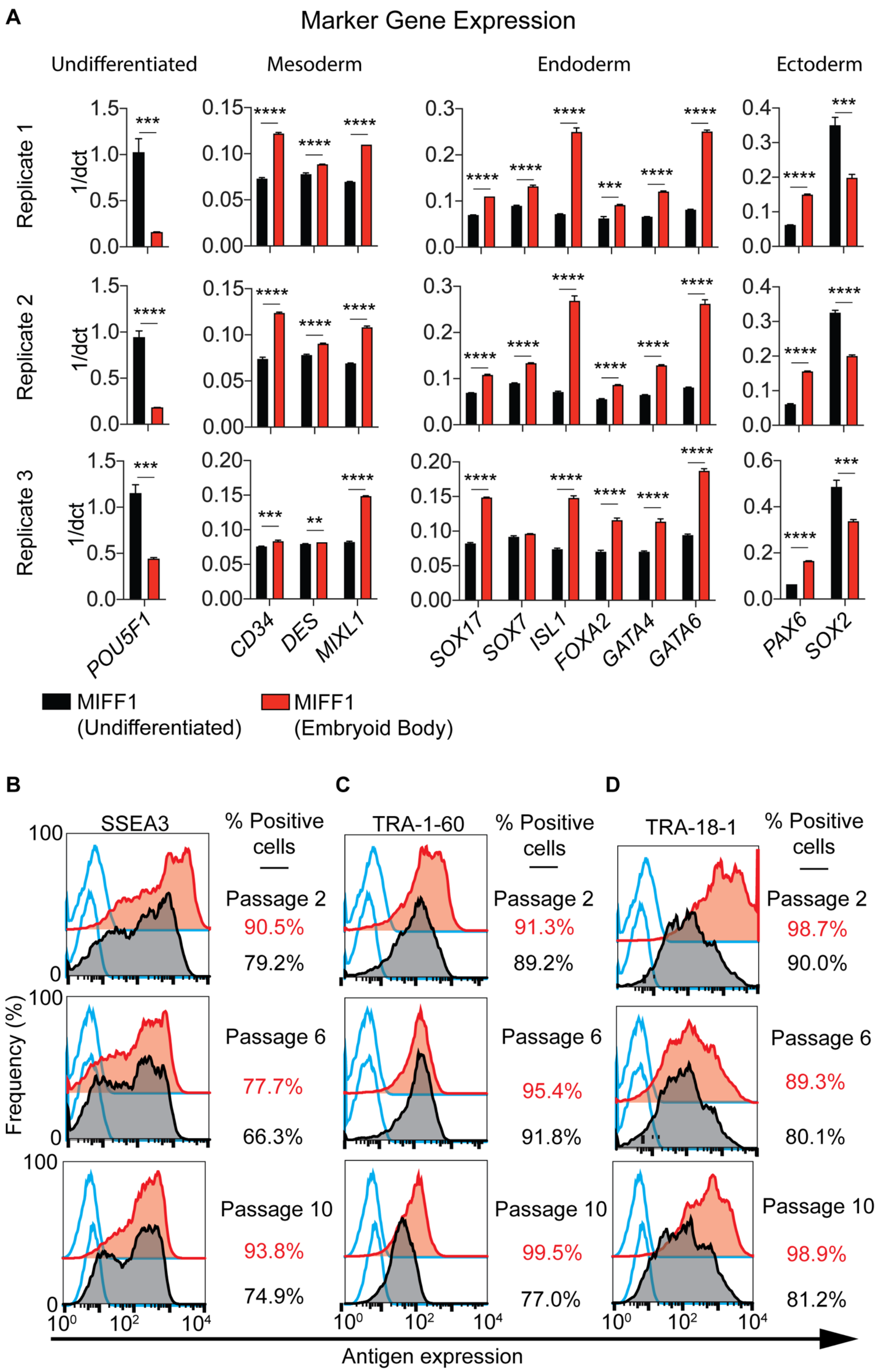
A
Marker Gene Expression
Undifferentiated
Mesoderm
Endoderm
Ectoderm
Replicate 1
Replicate 2
Replicate 3
1/dct
POU5F1
CD34
DES
MIXL1
SOX17
SOX7
ISL1
FOXA2
GATA4
GATA6
PAX6
SOX2
MIFF1 (Undifferentiated)
MIFF1 (Embryoid Body)
B
SSEA3
% Positive cells
Passage 2
90.5%
79.2%
Passage 6
77.7%
66.3%
Passage 10
93.8%
74.9%
C
TRA-1-60
% Positive cells
Passage 2
91.3%
89.2%
Passage 6
95.4%
91.8%
Passage 10
99.5%
77.0%
D
TRA-18-1
% Positive cells
Passage 2
98.7%
90.0%
Passage 6
89.3%
80.1%
Passage 10
98.9%
81.2%
Frequency (%)
Antigen expression

Figure S3 | Human PSC retain pluripotency associated antigen expression and the ability to differentiate into the three germ layers when cultured in exogenous nucleosides, additional data to support figure 3. A, Embryoid bodies were produced by differentiation under neutral conditions for 10 days from MIFF1 cells cultured with exogenous nucleosides for ten passages prior to differentiation. RT-qPCR analysis of marker gene expression by the embryoid bodies compared to gene expression in undifferentiated MIFF1 cells from which the EBs were derived. Genes associated with undifferentiated cells, mesoderm**,** endoderm and ectoderm are displayed. Each row shows the results from an individual biological replicate. Data of marker gene expression are means ± s.d., two-tailed *t*-test, *<P0.05, **P<0.01, ***P<0.001, ****P<0.0001 (*n* = 3 technical replicates per experiment). **B-D,** Pluripotency associated antigen expression, human PSC are grown with exogenous nucleosides (+ NUC) (Red) versus the same stem cell culture media in the absence of exogenous nucleosides (- NUC) (Black) over 10 passages. Baseline fluorescence was set using the primary antibody control, P3X (Blue). Percentage positive population is displayed to the right of each histogram. **B,** SSEA3 antigen expression from passage 2 to passage 10 (top to bottom). **C,** TRA-1-60 antigen expression passage 2 to 10. **D,** Tra-18-1 antigen expression from passage 2 to passage 10. **B-D,** Modal scaled channels, histograms are displayed as a percentage of the maximum count.

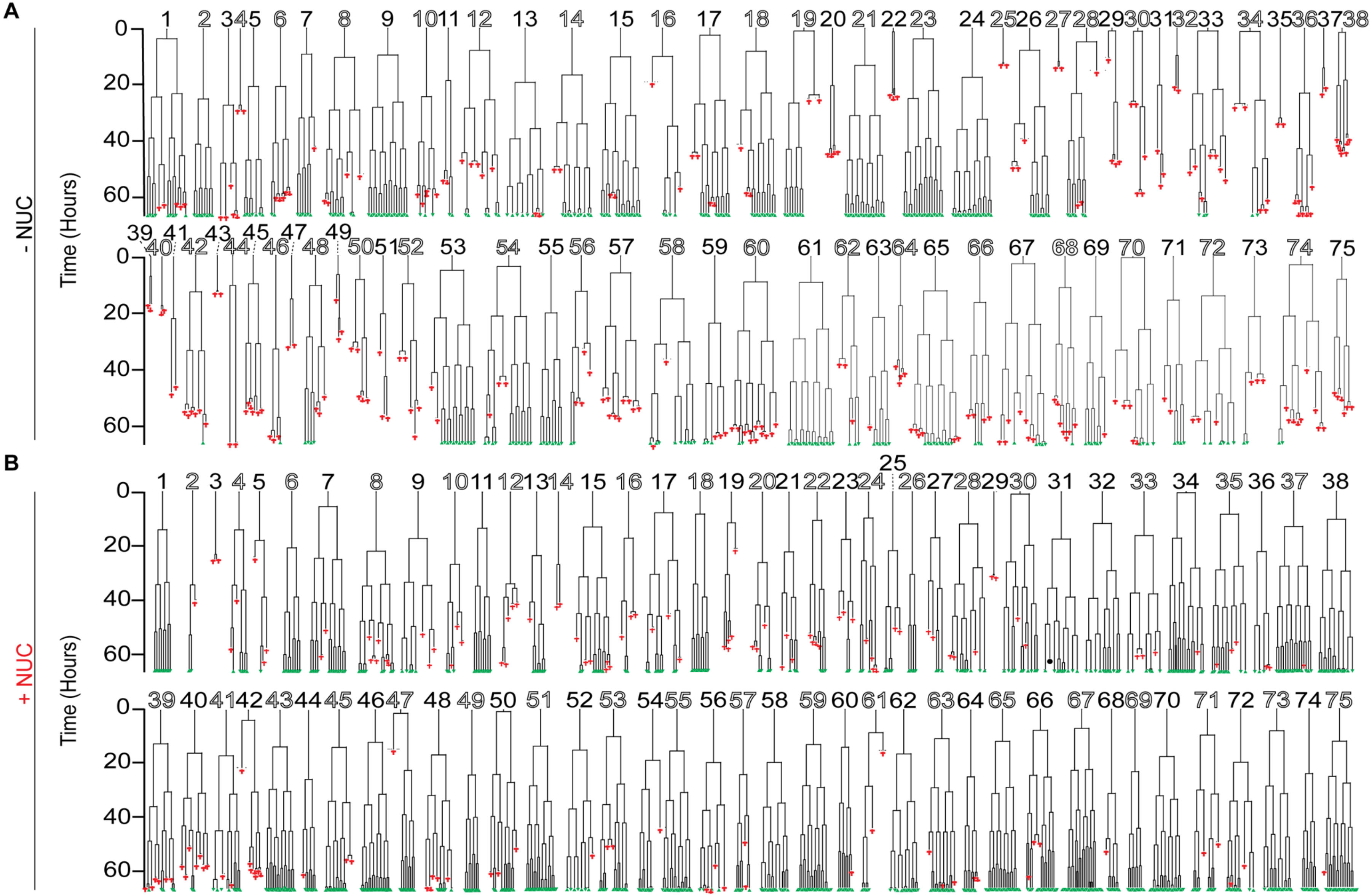
A
- NUC
Time (Hours)
0
20
40
60
1 2 3 4 5 6 7 8 9 10 11 12 13 14 15 16 17 18 19 20 21 22 23 24 25 26 27 28 29 30 31 32 33 34 35 36 37 38
39 40 41 42 43 44 45 46 47 48 49 50 51 52 53 54 55 56 57 58 59 60 61 62 63 64 65 66 67 68 69 70 71 72 73 74 75
B
+ NUC
Time (Hours)
1 2 3 4 5 6 7 8 9 10 11 12 13 14 15 16 17 18 19 20 21 22 23 24 25 26 27 28 29 30 31 32 33 34 35 36 37 38
39 40 41 42 43 44 45 46 47 48 49 50 51 52 53 54 55 56 57 58 59 60 61 62 63 64 65 66 67 68 69 70 71 72 73 74 75

Figure S4 | Exogenous nucleosides improve survival and plating efficiency of human PSC, figure S4 displays data from figure 4a in full. A,B, Lineage trees of the 75 cells, that reached the first cell division, sampled from MIFF1 grown in the absence of exogenous nucleosides (- NUC) (**A**) and when grown in the presence of exogenous nucleosides (+ NUC) (**B**) conditions. Twenty randomly selected starting cells and the resulting lineage trees are displayed in Figure 4.

Supplemental Experimental Procedures

Immunocytochemistry. Cells were fixed with 4% paraformaldehyde (Sigma, 158127) for 10 minutes. Cells were permeabilised and blocked with 0.3% Triton-X (Sigma, T8787), 10% goat serum (Thermo Fisher Scientific, 16210072) and 3% BSA (Sigma) in PBS for 1 hour. Primary antibody incubation was performed overnight at 4°C: Anti-Phospho-Histone H2A.X (Ser139) (Cell Signalling Technologies, 9718; 1:400), Anti-gamma H2A.X (Phospho S139) (abcam, ab26350; 1:500), Anti-Nanog (Cell Signalling, 4903; diluted 1:500), Anti-Nanog (Cell Signalling, 4893; diluted 1:500) and Anti-Ki67 (abcam, ab238020; diluted 1:100). Secondary antibody incubation was performed for 1 hour: Alexa Fluor 488-conjugated anti-rabbit IgG (Life Technologies, A11034; diluted 1:400), Alexa Fluor 647 AffiniPure Goat anti-Rabbit IgG (H+L) (Jackson Immuno Research, 111-605-003; 1:1000) and Alexa Fluor 647 AffiniPure Goat anti-Mouse IgG (H+L) (Jackson Immuno Research, 115-605-003; 1:400). All antibodies were diluted in 1% BSA (Sigma) and 0.3% Triton-X (Sigma, T8787) in PBS. Nuclei were counterstained with Hoechst 33342 (Thermo Fisher Scientific, H3570; diluted 1:1000) and images were acquired using an INCell Analyzer 2200 (GE Healthcare) high content microscope taking 25 or 30 randomized images per well.

Immunofluorescence data analysis. Where possible, CellProfiler(Carpenter et al., 2006) cell image analysis software was used to analyse the high content images. Immunofluorescence was quantified above a threshold set by a secondary only control. Hoechst 33342 was used to identify the cells nuclei and mask the nuclear area over the immunofluorescence staining. Where it was necessary to separate cells by cell cycle stage the integrated intensity of the stained nuclei was calculated using CellProfiler Analyst (Jones et al., 2008).

Neutral comet assay. 150μL of 0.6% agarose (Sigma, A9539) was set on a fully frosted glass slide, sandwiched beneath a coverslip. Once dried, 12,000 cells per conditions was resuspended in 75μL ice cold PBS and mixed with 75μL of 1.2% low melting agarose (Sigma, A4018). The cell and agarose suspension was mounted on top of the original agarose layer beneath a coverslip and set at 4°C. The slides were immersed in pre-chilled lysis buffer (2.5M NaCl, 10mM Tris-HCL, 100mM EDTA pH8.0, 0.5% Triton-X, 3% DMSO) for 1.5 hours at 4°C, washed in H_2O and equilibrized in electrophoresis buffer (300mM sodium acetate, 100mM Tris-EDTA and 1% DMSO) for 1 hour. Electrophoresis is performed at 25V for 1 hour. Slides were stained with SYBR green (Sigma, S9430), imaged and quantified using Comet Assay IV (Instem) live video measurement system.

DNA fibre assay. DNA fibre assay was performed as previously described (Groth et al., 2010). Briefly, cells were plated and grown for a minimum of 72 hours before sequential pulse labelling with 2.5mM CldU (Sigma, C6891; 1:100) and then 2.5mM IdU (Sigma, I7125; 1:10) for 20 minutes each. Cells were washed with ice cold PBS, dissociated using TrypLE cell dissociation enzyme (Thermo Fisher Scientific, 12504013) and diluted to $4x10^5$ cells/ml in cold PBS. To spread the labelled fibers, 2μL of cell suspension was dropped onto a glass slide and allowed to dry for 5-7 minutes before adding 7μL of spreading buffer (200mM Tris-HCL PH7.4, 50mM EDTA, 0.5% SDS). The solutions were mixed with a pipette tip and incubated for 2 minutes. Slides were tilted at an angle of 25° and the droplet timed to ensure consistent spreading was achieved. Slides were air dried and fixed with 3:1 methanol/acetic acid. For the immunostaining, the glass slides were first washed twice with H_2O for 5 minutes each, denatured with 2.5M HCL for 1 hour and then blocked in 1% BSA (Sigma) and 0.1% Tween20. Primary antibodies were incubated for 1 hour: Rat anti-BrdU, clone BU1/75 (Novus Biologicals NB500-169) (AbD Serotec; diluted 1:400) or Anti-BrdU clone BU1/75 (ICR1) (abcam, ab6326; diluted 1:400) and Mouse anti-BrDU (Clone B44) (Becton Dickinson, 347580; diluted 1:250). The secondary antibodies used were Alexa Fluor 555 goat anti-rat IgG (Thermo Fisher Scientific, A21434; diluted 1:500) and Alexa Fluor 488 F (ab')2-Goat anti-Mouse IgG (Thermo Fisher Scientific, A-11017; diluted 1:500). Slides were mounted with Fluoroshield (Sigma, F6182), and images were acquired using Olympus FV1000 confocal microscope.

RNA Extraction and reverse transcriptase qPCR. RNA was extracted using Qiagen RNeasy kit. cDNA synthesis was performed using high capacity reverse transcription kit (Thermo Fisher Scientific, 4368814). qPCR was performed in 384 well plates with 10μL reactions consisting of 1X TaqMan Fast Universal Master Mix (ThermoFisher, 4352042), 100nM of forward and reverse primers (Table S1), 100nM of probe from the Universal Probe Library (Roche) and 2μL of 5ng/μL cDNA. PCR reactions were run on the QuantStudio 12K Flex Thermocycler (Life Technologies 4471087). All reactions were performed in triplicate with comparative Ct normalized to GAPDH or B-ACTIN expression.

Table S1. Primer sequences and Universal probe library probes used in this study that relate to Figure 2, S2 and S6.

Gene	Sense	Anti-sense	Probe
POU5F1	agcaaaacccggaggagt	ccacatcggcctgtgtatatc	35
NANOG	agatgcctcacacggagact	tttgcgacactcttctctgc	31
SOX17	cgccgagttgagcaagat	ggtggtcctgcatgtgct	13
TFAP2A	acatgctcctggctacaaaac	aggggagatcggtcctga	62
TH	tcagtgacgccaaggaca	gtacgggtcgaacttcacg	42
NEUROD1	acctcgaagccatgaacg	cttccaggtcctcatcttcg	55
SOX7	ttcctcaccagccaggtc	atttgcgggaagttgctcta	30
AFP	tgtactgcagagataagtttagctgac	tccttgtaagtggcttcttgaac	61
FOXA2	cgccctactcgtacatctcg	agcgtcagcatcttgttgg	9
GATA6	aatacttcccccacaacacaa	ctctcccgcaccagtcat	90
MIXL1	gacacagatgaggggcagtt	cccgttttcagctaccattc	6
BRACHYURY	aggtacccaaccctgagga	gcaggtgagttgtcagaataggt	23
DES	ggagattgccacctaccg	ggtctggatggggagattg	55
PECAM1	ggtctggatggggagattg	ttcaagtttcagaatatcccaatg	37
GAPDH	agccacatcgctcagacac	gcccaatacgaccaaatcc	60
B-ACTIN	ccaaccgcgagaagatga	ccagaggcgtacagggatag	64
CCND2	agctgctggctaagatcacc	acggtctgctgcaggctat	68
CCNE1	ggccaaaatcgacaggac	catcatcttctttgtcaggtgtg	32

Western blotting. Laemili buffer (4% SDS, 20% Glycerol, 0.125M Tris HCl, 0.004% bromphenol blue) was added to cell pellets and sonicated for 10 seconds. Protein lysate was incubated for 10 minutes at 95°C and concentration was determined by NanoDrop spectrophotometer (Thermo Fisher Scientific).
Protein was separated on 10% ProtoGel (National Diagnostics) run at 120V for 1.5 hours and transferred onto PVDF membrane (Millipore, #IPVH00010). Primary antibodies were incubated over night at 4°C: α-Tubulin (Cell Signalling Technology, 2144; diluted 1:1000), Cyclin E1 (D7T3U) (Cell Signalling Technology, 20808; diluted 1:500), Cyclin D2 (D52F9) (Cell Signalling Technology, 3741; diluted 1:500). The blot was washed and incubated with Anti-rabbit IgG or anti-mouse IgG secondary antibody for 1 hour (Promega, W401 & W402). Immunoreactivity was visualised with ECL prime (GE Healthcare, RPN2232) on a CCD-based camera.

The generation of hPSC stably expressing H2B-RFP. Transfection with pCAG-H2B-RFP-IRES-PURO vector (Liew et al., 2007) was performed using electroporation. Cells were electroporated with a single 1600V pulse for 20msec using the Neon transfection system according to manufacturer's instructions (Thermo Fisher Scientific, MPK10025). Stable clones were obtained by puromycin selection. Puromycin concentration was increased gradually over 5 days to a final concentration of 0.375μg/mL before flow sorting for the brightest population of RFP.

Supplemental References

CARPENTER, A. E., JONES, T. R., LAMPRECHT, M. R., CLARKE, C., KANG, I. H., FRIMAN, O., GUERTIN, D. A., CHANG, J. H., LINDQUIST, R. A., MOFFAT, J., GOLLAND, P. & SABATINI, D. M. 2006. CellProfiler: image analysis software for identifying and quantifying cell phenotypes. *Genome Biol,* 7**,** R100.

GROTH, P., AUSLÄNDER, S., MAJUMDER, M. M., SCHULTZ, N., JOHANSSON, F., PETERMANN, E. & HELLEDAY, T. 2010. Methylated DNA causes a physical block to replication forks independently of damage signalling, O(6)-methylguanine or DNA single-strand breaks and results in DNA damage. *J Mol Biol,* 402**,** 70-82.

JONES, T. R., KANG, I. H., WHEELER, D. B., LINDQUIST, R. A., PAPALLO, A., SABATINI, D. M., GOLLAND, P. & CARPENTER, A. E. 2008. CellProfiler Analyst: data exploration and analysis software for complex image-based screens. *BMC Bioinformatics,* 9**,** 482.

LIEW, C. G., DRAPER, J. S., WALSH, J., MOORE, H. & ANDREWS, P. W. 2007. Transient and stable transgene expression in human embryonic stem cells. *Stem Cells,* 25**,** 1521-8.
