# Supplementary material for: Nucleosides Rescue Replication-Mediated Genome Instability of Human Pluripotent Stem Cells
Source: Stem Cell Reports. 2020 May 14;14(6):1009–17. doi: 10.1016/j.stemcr.2020.04.004 (PMC7355123; doi:10.1016/j.stemcr.2020.04.004)
Supplement: Document S2. Article plus Supplemental Information [file mmc2.pdf]

Stem Cell Reports
Report

OPEN ACCESS

Nucleosides Rescue Replication-Mediated Genome Instability of Human Pluripotent Stem Cells

Jason A. Halliwell,[1] Thomas J.R. Frith,[1] Owen Laing,[1] Christopher J. Price,[1] Oliver J. Bower,[1] Dylan Stavish,[1] Paul J. Gokhale,[1] Zoe Hewitt,[1] Sherif F. El-Khamisy,[2] Ivana Barbaric,[1,*] and Peter W. Andrews[1,*]
[1]Department of Biomedical Science, University of Sheffield, Western Bank, Sheffield S10 2TN, UK
[2]Krebs Institute, Department of Molecular Biology and Biotechnology, University of Sheffield, Sheffield S10 2TN, UK
*Correspondence: i.barbaric@sheffield.ac.uk (I.B.), p.w.andrews@sheffield.ac.uk (P.W.A.)
https://doi.org/10.1016/j.stemcr.2020.04.004

SUMMARY

Human pluripotent stem cells (PSCs) are subject to the appearance of recurrent genetic variants on prolonged culture. We have now found that, compared with isogenic differentiated cells, PSCs exhibit evidence of considerably more DNA damage during the S phase of the cell cycle, apparently as a consequence of DNA replication stress marked by slower progression of DNA replication, activation of latent origins of replication, and collapse of replication forks. As in many cancers, which, like PSCs, exhibit a shortened G1 phase and DNA replication stress, the resulting DNA damage may underlie the higher incidence of abnormal and abortive mitoses in PSCs, resulting in chromosomal non-dysjunction or cell death. However, we have found that the extent of DNA replication stress, DNA damage, and consequent aberrant mitoses can be substantially reduced by culturing PSCs in the presence of exogenous nucleosides, resulting in improved survival, clonogenicity, and population growth.

INTRODUCTION

Human pluripotent stem cells (PSCs) often acquire genetic changes on prolonged culture, which pose concerns for their use in research and regenerative medicine (Amps et al., 2011; Taapken et al., 2011). The acquisition of these changes during culture necessarily first requires mutation and then selection of those mutations that provide a growth advantage. Whereas selection accounts for the recurrent nature of the variants commonly reported (Draper et al., 2004; Olariu et al., 2010), the mechanisms of acquired mutation in PSCs remain largely unexplored. Recent work has started to elucidate environmental conditions that have an impact on the mutational burden of PSCs, highlighting oxidative stress as a major contributor to the acquisition of single nucleotide variation in PSCs (Thompson et al., 2020). In addition to such extrinsic influences, intrinsic properties of PSCs are also thought to play a role in the maintenance of their genome stability (Ahuja et al., 2016). The defining features of PSCs that make them distinct from somatic cells are the pluripotent gene regulatory network responsible for maintaining the pluripotent state (Pera et al., 2000), and a rapid cell cycle due to an abbreviated G1 compared with somatic cells (Becker et al., 2006). In mouse embryonic stem cells (ESCs), the abbreviated G1 was shown to cause replication stress (Ahuja et al., 2016), a collective term for interruption of the DNA replication process, which manifests as a slowing or stalling of the DNA replication fork, resulting in DNA breaks upon a fork collapse (Zeman and Cimprich, 2014). Replication stress due to an abbreviated G1 is also implicated in cancer development, wherein high levels of cyclin E drive the premature entry of cells into the S phase in the absence of sufficient nucleotide pools needed for normal progression of the replication fork (Bester et al., 2011). Consequently, the nucleotide-deficient cells exhibit replication stress, predisposing them to DNA damage and subsequent genome instability (Bester et al., 2011; Burrell et al., 2013).

A high level of DNA damage in human PSCs has been reported previously (Simara et al., 2017; Vallabhaneni et al., 2018), but the underlying causes and means of alleviating the damage remain unknown. Here, we show that an increased susceptibility to DNA damage in PSCs compared with their isogenic differentiated cells is caused by persistent replication stress in PSCs. Importantly, we demonstrate that the addition of exogeneous nucleosides to the culture medium restores the replication dynamics and reduces the level of genome damage and incidence of mitotic aberrations in PSCs. Finally, we show that nucleoside supplementation also improves survival of PSCs, demonstrating that replication stress-associated genome damage is a major cause of cell death in PSC cultures.

RESULTS AND DISCUSSION

DNA double-strand breaks (DSBs) are a particularly detrimental type of DNA damage. Unrepaired, or erroneously repaired, DSBs can jeopardize genome stability by leading to mitotic aberrations and structural and chromosomal instability (Ichijima et al., 2010; Janssen et al., 2011), like those frequently observed in human PSCs (Amps et al., 2011). The human induced PSC line MIFF1 (Desmarais et al., 2016) exhibited an increased number of γH2AX

This is an open access article under the CC BY license (http://creativecommons.org/licenses/by/4.0/).

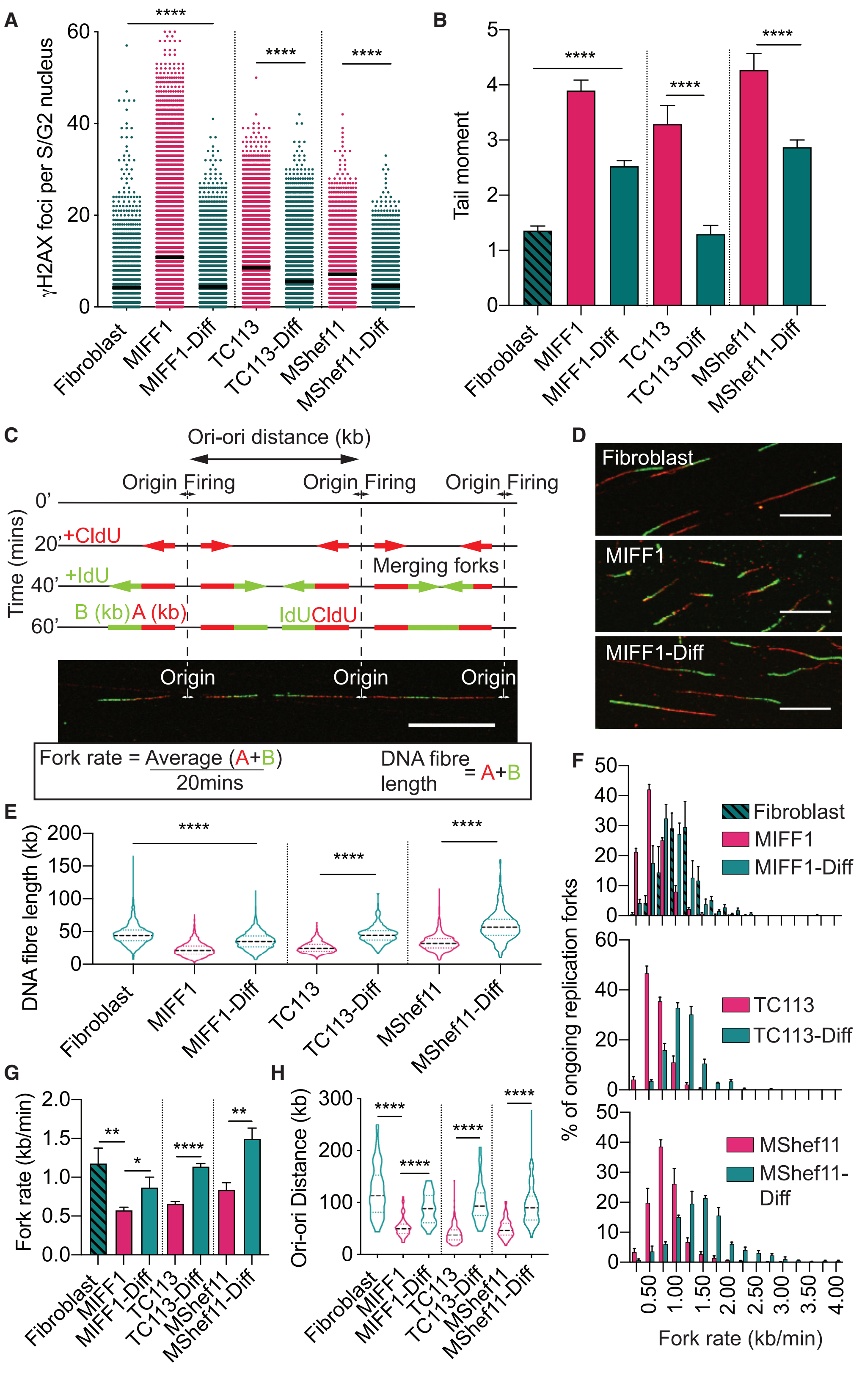

(legend on next page)

foci (Figure 1A), known to mark sites of DSBs, compared with the fibroblast from which it was reprogrammed and to its differentiated derivatives, obtained by treatment with CHIR99021 for 5 days (Figure S1). Two other PSC lines that we examined, TC113 (Baghbaderani et al., 2015) and MShef11 (Thompson et al., 2020), showed a similarly increased level of DNA damage compared with their differentiated derivatives (Figures 1A and S1). These observations were confirmed by a neutral comet assay that showed an increased tail moment in the undifferentiated cells in each case (Figure 1B). We also compared the growth of MIFF1 in four commercially available feeder-free media and found similar levels of γH2AX foci, although slightly lower in cells grown in Nutristem (Figure S2A). Overall, the results demonstrate that a high level of genome damage is associated with the pluripotent state and decreases upon differentiation.

A common cause of DSBs during S phase in cancer cells is the slowing, stalling, and collapse of replication forks, recognized as DNA replication stress (Ichijima et al., 2010). To analyze the replication dynamics in undifferentiated and differentiated cells, we utilized the DNA fiber assay. Here, the newly synthesized DNA is pulse labeled successively with thymidine analogs, cholorodeoxyuridine (CldU) and iododeoxyuridine (IdU), for 20 min each and then visualized by fluorescently labeled antibodies (Figure 1C). By measuring the total length of the CldU and IdU labeling in each fiber, we found a decrease in the length of newly synthesized fibers in the undifferentiated state (Figures 1C–1E). Replication fork speed, calculated by measuring the average length of labeled fibers, was significantly slower in undifferentiated PSCs compared with their isogenic somatic counterparts (Figures 1C, 1F, and 1G). Further, we found an increase in the abundance of origins of DNA replication as demonstrated by a decrease in replication origin-to-origin distance (Ori-ori) in PSCs (Figures 1C and 1H). Overall, these results show that DNA replication in pluripotent cells is considerably perturbed, predisposing them to DNA damage, notably DSBs.

The association of genome damage with the pluripotent rather than the somatic state of the same cell line, suggests that features linked to pluripotency impart replication stress on PSCs. A pertinent key difference between the pluripotent and somatic cell state is the rapid progression of PSCs through G1, driven by atypical expression of cyclins (Becker et al., 2006; Ahuja et al., 2016). By time-lapse microscopy and 5-ethynyl-2′-deoxyuridine (EdU) pulse-chase analysis, we found that the human PSC line, MIFF1, exhibited a reduced cell-cycle time when compared with its parent fibroblast line (Figure 2A). Specifically, the abbreviated cell-cycle time was solely due to a truncated G1 (Figure 2A). Consistent with the reduction in the length of G1, cyclin D2 (*CCND2*) and cyclin E (*CCNE1*), which are known to drive the Rb-E2F pathway and allow rapid progression of cells through G1 (Hinds et al., 1992; Lundberg and Weinberg, 1998), were highly expressed in the undifferentiated MIFF1 compared with the corresponding parent fibroblast line (Figures 2B–2E). We confirmed these findings in TC113 and MShef11, which also exhibited a similar short G1 and enhanced expression of cyclin D2 and E (Figures 2A–2C).

We reasoned that the short G1 may have an impact on genome damage of PSCs, since overexpression of cyclin D2 and E in cancer cells has also been reported to enforce an abbreviation of G1 and consequent replication stress, which can be modulated by exogenous nucleosides (Bester et al., 2011; Takano et al., 2000). We tested whether the addition of exogenous nucleosides would improve the

Figure 1. Replication Stress and a Susceptibility to DNA Damage is a Characteristic of Undifferentiated Human PSCs
Data in the figure compare undifferentiated and differentiated cell states. MIFF1 was compared with its parent fibroblast line (Fibroblast) and with its differentiated derivative (MIFF1-Diff). TC113 and MShef11 were compared with their differentiated derivatives (TC113-Diff and MShef11-Diff).
(A) The number of γH2AX foci per S-/G2-phase cell. The S/G2 phase was determined from nuclear DNA content. Data points in (A) represent individual cells and are the results from three independent experiments; the center line indicates the mean. Two-tailed t test, ****$p < 0.0001$ ($n > 100$ cells per cell line per experiment).
(B) Average tail moment from neutral comet assays. Data displayed are from three independent experiments ± SEM. Two-tailed t test, ****$p < 0.0001$ ($n \geq 300$ cells per cell line per experiment).
(C) Schematic of DNA fiber analysis. Sequential 20-min pulses of CldU and IdU labeled the progressing replication forks. Measurements of CldU and IdU lengths enable the analysis of replication fork dynamics. Scale bar, 10 μm.
(D) Representative DNA fibers are shown for Fibroblast, MIFF1, and MIFF1-Diff. Scale bar, 10 μm.
(E) Combined length of CldU and IdU in individual fibers ($n > 200$ forks per cell line per experiment, n = 3 experiments). Median distance, 25th and 75th quartiles are presented. Two-tailed t test, ****$p < 0.0001$.
(F) Distribution of replication fork rates ($n > 200$ forks per cell line per experiment, n = 3 experiments). Data are mean values from each experiment ± SEM.
(G) Mean fork rates from (F) ± SD. Two-tailed *t* test, *$p < 0.05$, **$p < 0.01$, ****$p < 0.0001$ (n = 3 experiments).
(H) Distribution of adjacent origins distance measurements (Ori-ori). Median distance, 25th and 75th quartiles are presented. Two-tailed t test, ****$p < 0.0001$ ($n > 30$ per cell line, n = 3 experiments).

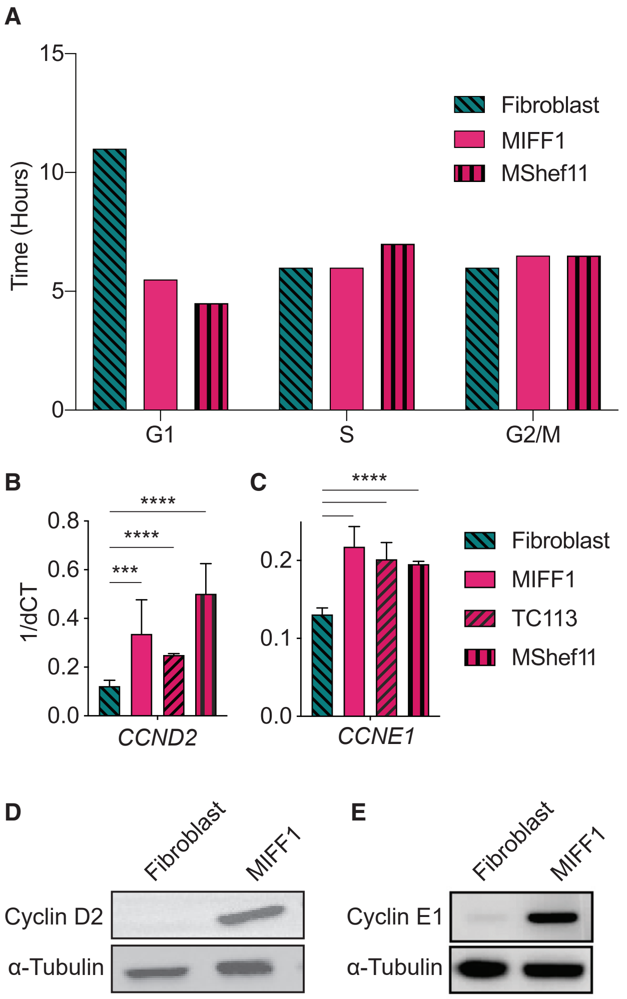

Figure 2. Cell-Cycle Dynamics and Cyclin Expression in Human PSCs Are Candidates for Replication Stress Initiation

(A) Cell-cycle phase time determined from time-lapse microscopy (Fibroblast, n = 44; MIFF1, n = 76; MShef11, n = 31) and EdU pulse-chase analysis of asynchronous cells (minimum of 10,000 events recorded during fluorescence-activated cell sorting analysis).

(B and C) RT-qPCR gene expression data from MIFF1, TC113, and MShef11 for *CCND2* (B), *CCNE* (C). Data in (B) and (C) are means ± SD. Two-tailed t test, ***$p < 0.001$, ****$p < 0.0001$ (n = 3 experiments).

(D and E) Representative western blot of protein expression for cyclin D2 (D), cyclin E1 (E).

replication dynamics of human PSCs. After an initial titration of nucleosides, using γH2AX as a readout of genome damage, we chose a formulation 15μM cytidine, 15μM guanosine, 15μM uridine, 15μM adenosine, and 6μM thymidine. The addition of these exogenous nucleosides increased DNA fiber lengths and replication fork speed in MIFF1 to levels comparable with those observed in its differentiated derivatives (Figures 3A–3D compared with Figures 1D–1G). In addition, we noted fewer CldU-only tracts, indicating fewer forks stalled prior to the addition of the second thymidine analog, IdU (Figure 3E). There was also a decrease in replication origin density, with Ori-ori distances in MIFF1 now comparable with those observed in the parent fibroblast line and following the differentiation of MIFF1 (Figure 3F compared with Figure 1H), suggesting that, as a consequence of slower fork speed, the cells were firing from dormant origins in the absence of exogenous nucleosides. We confirmed these findings in TC113 and MShef11, which show a similar increase in replication fork speed and Ori-ori distance with the addition of exogenous nucleosides (Figures S2B–S2F). Under these conditions we observed a marked decrease in the frequency of DSBs in MIFF1, TC113, and MShef11 as indicated by a reduction in the number of γH2AX foci per S-phase and G2-phase cells upon addition of nucleosides (Figures 3G and S2G) and a decrease in tail moment measured using the neutral comet assay (Figure 3H). Similarly, exogenous nucleosides also reduced the number of γH2AX foci when the cells were cultured in alternative human PSC culture media, Nutristem and E8 (Figure S2H), while also retaining the ability to differentiate (Figure S3). Overall, these results indicate that susceptibility to DNA damage observed in the undifferentiated PSCs is a consequence of replication stress that can be alleviated by exogenous nucleosides.

A detrimental consequence of replication stress is the presence of under-replicated regions that can persist into mitosis and hinder chromosome separation (Bester et al., 2011; Burrell et al., 2013; Ichijima et al., 2010). This in turn can lead to mitotic aberrations, including chromosome bridges, lagging chromosomes, and the formation of micronuclei (Crasta et al., 2012; Ichijima et al., 2010; Janssen et al., 2011). Using time-lapse microscopy of MIFF1 cells stably transfected with H2B-RFP to fluorescently label chromatin, we tracked the progression of cells through mitosis. Consistent with previous reports (Lamm et al., 2016; Zhang et al., 2019), we observed a high incidence of mitotic errors in human PSCs. However, the incidence of these errors was significantly decreased in cells cultured in the presence of nucleosides (Figures 3I and 3J), indicating that replication stress in human PSCs is a cause of mitotic errors.

To investigate the consequences of these observations for the proliferation of human PSCs, we used time-lapse microscopy to track the growth of single cells through successive divisions. When MIFF1 cells were seeded at low density, 68% of those that attached went on to divide in normal culture medium, whereas 79% entered mitosis in medium supplemented with nucleosides. This is consistent with our previous observation that human PSCs activate apoptosis in response to replication stress (Desmarais

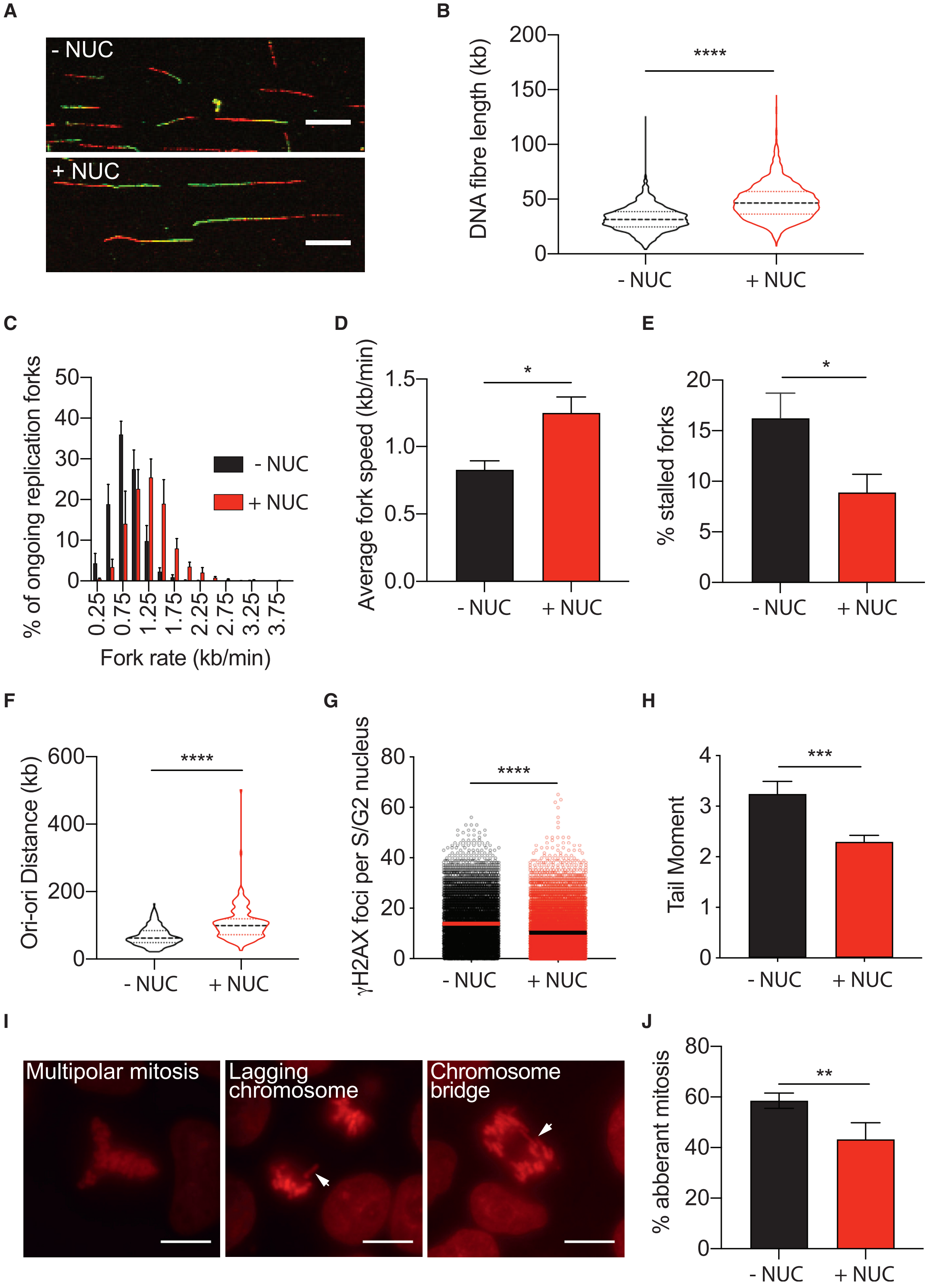

(legend on next page)

et al., 2012, 2016). Of those that did enter mitosis, 59% went on to form colonies of two or more cells under standard culture conditions, with many cells dying after the first and subsequent divisions (Figures 4A and S4A). By contrast, fewer cells died following division when exogenous nucleosides were added and 91% of cells went on to form colonies (Figures 4A and S4B) with substantially greater final size (Figure 4B). In the absence of added nucleosides, there was a consistently higher number of abortive cell divisions involving the death of both daughter cells (Figures 4C and 4D), a result that would be anticipated if the mitotic errors caused by DNA replication stress are catastrophic for both daughter cells.

Taken together, our results demonstrate that human PSCs, compared with somatic cells, are predisposed to high levels of replication stress, manifest by slower rates of DNA synthesis, activation of latent origins of replication, and the stalling of replication forks. One consequence is their susceptibility to double-stranded DNA breaks that, in turn, may lead to genomic rearrangements during mitosis (Ichijima et al., 2010; Janssen et al., 2011). These results reflect the requirement for rapid replication of human PSCs enabled by a truncated G1 (Becker et al., 2006, 2010) that impairs the preparation of these cells for the ensuing DNA replication. However, a further feature of human PSCs is that, unlike somatic cells, they tend to undergo apoptosis in response to replication stress, so minimizing the appearance of mutant cells. This might reflect the demands of cell proliferation in the early embryo in which any genomic damage in even one cell could be catastrophic for the whole embryo (Desmarais et al., 2012, 2016). Indeed, in a separate study, we have found that the overall mutation rate in human PSCs is rather low (Thompson et al., 2020), despite their propensity to DNA damage. Nevertheless, that human PSCs tend to accumulate particular recurrent mutations and genomic rearrangements, most likely reflects selection for growth advantages among those few variants that escape apoptosis. It is notable that resistance to apoptosis is a common feature of many recurrent variants that do arise in human PSCs (Avery et al., 2013; Barbaric et al., 2014; Merkle et al., 2017). Our observation that exogenous nucleosides substantially reduced DNA replication stress in human PSCs, perhaps compensating for metabolic changes that stem from their shortened G1 and relaxed G1/S transition, provides a means to reduce the incidence of recurrent genetic changes that may compromise the use of human PSCs for disease modeling and regenerative medicine.

EXPERIMENTAL PROCEDURES

Human Pluripotent Stem Cell Culture

Two human iPSC lines, MIFF1 and TC113, and one human ESC line, MShef11, were used in this study. MIFF1 had been reprogrammed at the University of Sheffield, Centre for Stem Cell Biology, from a human foreskin fibroblast, CCD-112Sk (ATCC, CRL-2429) using a vector-free mRNA reprogramming system (Desmarais et al., 2016) (hPSCreg: https://hpscreg.eu/cell-line/UOSi001-A). The second human iPSC line, TC113, was acquired from RUCDR Infinite Biologics. It had been reprogrammed from CD34+ umbilical cord blood cells using a non-integrating episomal vector reprogramming system (Baghbaderani et al., 2015) (hPSCreg: https://hpscreg.eu/cell-line/RUCDRi002-A). Finally, the human ESC line, MShef11, had been isolated at the University of Sheffield, Centre for Stem Cell Biology, under good manufacturing practice-like conditions in a cleanroom setting (HFEA license R115-8-A (Centre 0191) and HTA license 22510). The fresh embryo was a surplus/unsuitable embryo for *in vitro* fertilization treatment, donated from the assisted conception unit and cultured to the blastocyst stage using *in vitro* fertilization medium (Medicult) before explantation onto human feeders

Figure 3. Exogenously Supplied Nucleosides Alleviate Replication Stress in Human PSCs

(A) Representative images of DNA fibers in the absence of nucleosides (−NUC) and in the presence of exogenous nucleoside (+NUC) conditions. Scale bar, 10 μm.

(B) Combined length of CldU and IdU in individual fibers ($n > 200$ forks per cell line per experiment, $n = 3$ experiments). Median distance, 25th and 75th quartiles are presented. Two-tailed t test, ***$p < 0.001$.

(C) Distribution of replication fork rates ($n > 200$ forks per condition per experiment, $n = 3$ experiments). Data are means ± SEM.

(D) Average fork rates from (C). Data are means ± SD. Two-tailed t test, *$p < 0.05$ ($n = 3$ experiments).

(E) Frequency of CldU-only tracts that denote a stalled replication fork ($n > 700$ forks per condition per experiment, $n = 3$ experiments). Data are means ± SD. Two-tailed t test, *$p < 0.05$.

(F) Distribution of adjacent origins distance measurements (Ori-ori). Median distance, 25th and 75th quartiles are presented. Two-tailed t test, ****$p < 0.0001$ ($n > 150$ per cell line, $n = 3$ experiments).

(G) γH2AX foci per S-/G2-phase cell (determined from DNA content). Each data point is the measurement of an individual cell; the center line indicates the mean. Two-tailed t test, ****$p < 0.0001$ ($n > 100$ cells per condition per experiment, $n = 3$ experiments).

(H) Average tail moment from neutral comet assay experiments. Data are means ± SD. Two-tailed t test, ***$p < 0.001$ ($n \geq 300$ cells per condition per experiment, $n = 3$ experiments).

(I and J) Mitotic errors observed from fluorescently labeled chromatin (histone H2B-RFP). Representative images of mitotic errors (I). White arrows point to mitotic error in each case. Scale bar, 10 μm. Average frequency of mitotic errors observed (J). Data are means ± SD. Unpaired t test, **$p < 0.01$ ($n = 13$–43 mitosis assessed per condition per experiment, $n = 4$ experiments).

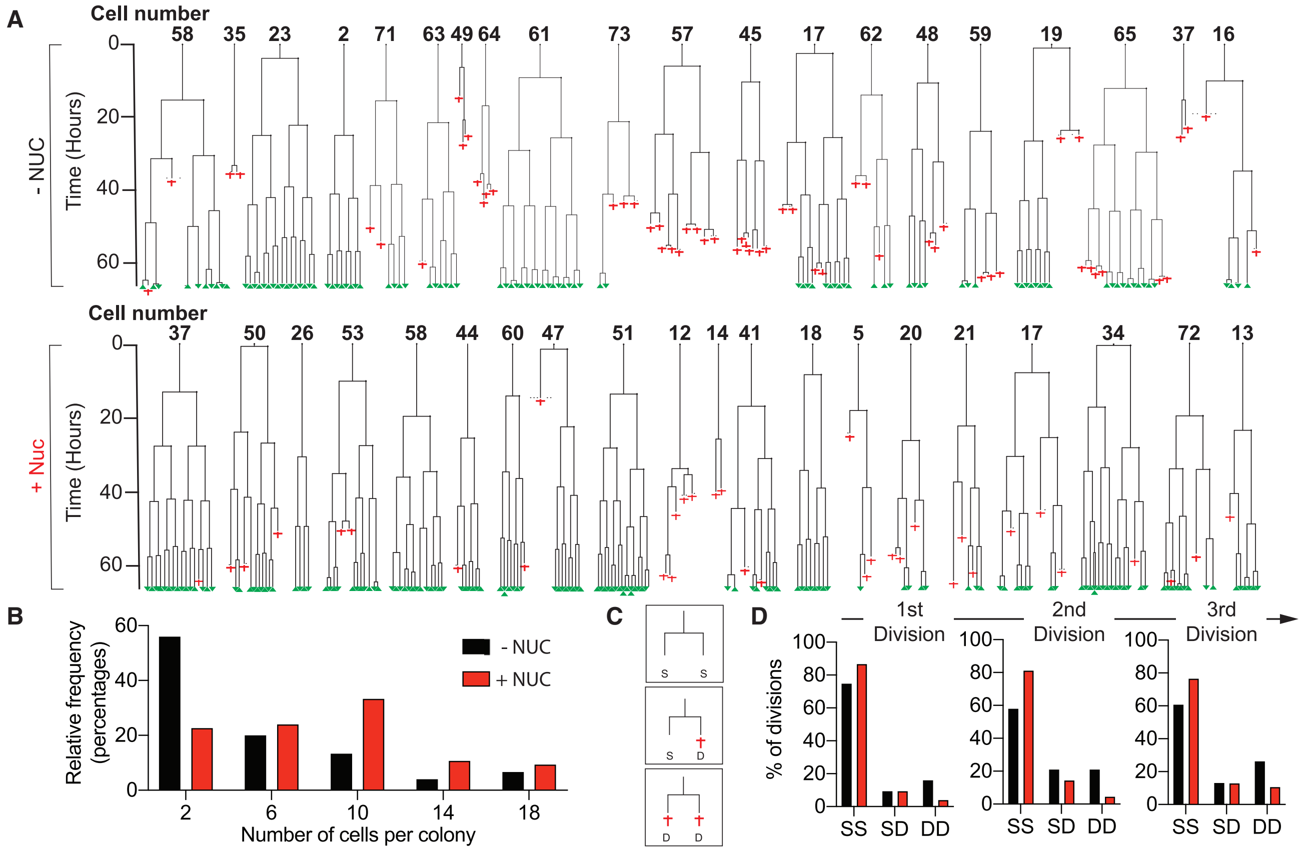

Figure 4. Exogenous Nucleosides Improve Survival and the Ability of Human PSCs to Re-enter the Cell Cycle Post Plating

(A) Time-lapse data tracing the growth of individual MIFF1 cells that reach the first cell division plotted as lineage trees. A random sample of 20 cells (full set of 75 cells shown in Figure S4) grown without exogenous nucleosides (top, −NUC) and in the presence of exogenous nucleosides (bottom, +NUC). Where the trees fork indicates a cell division, red crosses show cell death, and surviving cells at the end of the time lapse are noted with a green triangle. Time is displayed on the y axis.

(B–D) Summary data of the lineage tree analysis (B). Histogram of the distribution colony sizes at the end of the time-lapse experiment (C). Schematic illustrating the scoring method used in (D). After each division, the fate of the daughter cells was recorded: both daughter cells surviving (SS), one daughter cell survives and one dies (SD), or both daughter cells die (DD). (D) Individual bar charts show the frequency of SS, SD and DD daughter cell fates following the first, second and third divisions (left to right) after plating (D). Cell culture media without exogenous nucleosides (black) and grown in the presence of exogenous nucleosides (red) conditions are shown.

(Thompson et al., 2020) (hPSCreg: https://hpscreg.eu/cell-line/UOSe015-A).

These PSCs were all cultured on vitronectin (VTN-N) recombinant human protein (Thermo Fisher Scientific, A14700). Culture vessels were coated with 200μL/cm^2 of vitronectin that had been diluted to 6μg/mL with PBS and incubated at 37°C for at least 1 h. The cells were maintained in feeder-free conditions, batch fed daily with Essential 8 (Chen et al., 2011), mTeSR1 (STEMCELL Technologies, 85850), NutriStem XF (Biological Industries, 05-100-1A) or Essential 8 medium (Chen et al., 2011) without fibroblast growth factor 2 (FGF2) but supplemented with StemBeads FGF2 (800μL per 100 mL) (StemCultures, SB500), and incubated in a 37°C, 5% CO_2 humidified incubator. Passaging of cells was performed by clump dissociation using ReLeSR (STEMCELL Technologies, 05,873) following the manufacturer's guidelines.

Differentiation of Human PSCs

Human PSCs were grown for 5 days in E8 medium (Chen et al., 2011)without FGF2 and transforming growth factor β but supplemented with 10 μM CHIR99021 (Tocris, 4423). Loss of pluripotency was confirmed by an RT-qPCR panel of self-renewal, mesoderm, endoderm, and ectoderm genes (Table S1) and by immunofluorescence staining and imaging of NANOG.

Fibroblast Cell Culture

Fibroblasts (ATCC, CRL2429) were grown in Iscove's modified Dulbecco's medium (Thermo Fisher Scientific, 12440053) with 20% fetal bovine serum (HyCLone, SV30160.03). Cells were passaged using TrypLE cell dissociation enzyme (Thermo Fisher Scientific, 12504013) following the manufacturer's guidelines. Cells were maintained at 37°C and 5% CO_2 in a humidified incubator.

Nucleoside Supplementation

EmbryoMax Nucleosides 100X (Merck, ES-008-D) were added to mTeSR, E8, Nutristem, or StemBeads cell culture media at a final concentration of 0.5× (15μM cytidine, 15μM guanosine, 15μM uridine, 15μM adenosine, and 6μM thymidine). All experiments were performed after 72 h in culture with the supplementation of nucleosides.

SUPPLEMENTAL INFORMATION

Supplemental Information can be found online at https://doi.org/10.1016/j.stemcr.2020.04.004.

AUTHOR CONTRIBUTIONS

P.W.A. and I.B. oversaw the project. J.A.H., I.B., and P.W.A devised the experiments. J.A.H. performed most cell biology experiments with help from T.J.R.F., O.L., C.J.P., D.S., and O.J.B. J.A.H. and T.J.R.F. performed the differentiations of the human PSCs. J.A.H. and O.L. performed embryoid body formation and pluripotency-associated antigen expression analysis. J.A.H., C.J.P., D.S., and O.J.B. performed time-lapse lineage tree experimentation and analysis. Z.H. oversaw the derivation of the hiPSC2 cell line. P.W.A., I.B., P.J.G., and S.F.E. provided experimental advice. The manuscript was drafted by J.A.H., P.W.A., and I.B.

ACKNOWLEDGMENTS

This project has received funding from the European Union Horizon 2020 research and innovation program under grant agreement no. 668724. This work was partly funded by the European Union Horizon 2020 research and innovation program under grant agreement no. 668724 and partly by the UK Regenerative Medicine Platform, MRC reference MR/R015724/1.

Received: March 30, 2020
Revised: April 14, 2020
Accepted: April 14, 2020
Published: May 14, 2020

REFERENCES

Ahuja, A.K., Jodkowska, K., Teloni, F., Bizard, A.H., Zellweger, R., Herrador, R., Ortega, S., Hickson, I.D., Altmeyer, M., Mendez, J., and Lopes, M. (2016). A short G1 phase imposes constitutive replication stress and fork remodelling in mouse embryonic stem cells. Nat. Commun. *7*, 10660.

Amps, K., Andrews, P.W., Anyfantis, G., Armstrong, L., Avery, S., Baharvand, H., Baker, J., Baker, d., Munoz, M.B., Beil, S., et al. (2011). Screening ethnically diverse human embryonic stem cells identifies a chromosome 20 minimal amplicon conferring growth advantage. Nat. Biotechnol. *29*, 1132–1144.

Avery, S., Hirst, A.J., Baker, D., Lim, C.Y., Alagaratnam, S., Skotheim, R.I., Lothe, R.A., Pera, M.F., Colman, A., Robson, P., et al. (2013). BCL-XL mediates the strong selective advantage of a 20q11.21 amplification commonly found in human embryonic stem cell cultures. Stem Cell Rep. *1*, 379–386.

Baghbaderani, B.A., Tian, X., Neo, B.H., Burkall, A., Dimezzo, T., Sierra, G., Zeng, X., Warren, K., Kovarcik, D.P., Fellner, T., and Rao, M.S. (2015). cGMP-manufactured human induced pluripotent stem cells are available for pre-clinical and clinical applications. Stem Cell Rep. *5*, 647–659.

Barbaric, I., Biga, V., Gokhale, P.J., Jones, M., Stavish, D., Glen, A., Coca, D., and Andrews, P.W. (2014). Time-lapse analysis of human embryonic stem cells reveals multiple bottlenecks restricting colony formation and their relief upon culture adaptation. Stem Cell Rep. *3*, 142–155.

Becker, K.A., Ghule, P.N., Lian, J.B., Stein, J.L., Van Wijnen, A.J., and Stein, G.S. (2010). Cyclin D2 and the CDK substrate p220(NPAT) are required for self-renewal of human embryonic stem cells. J. Cell Physiol. *222*, 456–464.

Becker, K.A., Ghule, P.N., Therrien, J.A., Lian, J.B., Stein, J.L., Van Wijnen, A.J., and Stein, G.S. (2006). Self-renewal of human embryonic stem cells is supported by a shortened G1 cell cycle phase. J. Cell Physiol. *209*, 883–893.

Bester, A.C., Roniger, M., Oren, Y.S., Im, M.M., Sarni, D., Chaoat, M., Bensimon, A., Zamir, G., Shewach, D.S., and Kerem, B. (2011). Nucleotide deficiency promotes genomic instability in early stages of cancer development. Cell *145*, 435–446.

Burrell, R.A., Mcclelland, S.E., Endesfelder, D., Groth, P., Weller, M.C., Shaikh, N., Domingo, E., Kanu, N., Dewhurst, S.M., Gronroos, E., and Chew, S.K. (2013). Replication stress links structural and numerical cancer chromosomal instability. Nature *494*, 492–496.

Chen, G., Gulbranson, D.R., Hou, Z., Bolin, J.M., Ruotti, V., Probasco, M.D., Smuga-Otto, K., Howden, S.E., Diol, N.R., Propson, N.E., et al. (2011). Chemically defined conditions for human iPSC derivation and culture. Nature Methods *8*, 424–429.

Crasta, K., Ganem, N.J., Dagher, R., Lantermann, A.B., Ivanova, E.V., Pan, Y., Nezi, L., Protopopov, A., Chowdhury, D., and Pellman, D. (2012). DNA breaks and chromosome pulverization from errors in mitosis. Nature *482*, 53–58.

Desmarais, J.A., Hoffmann, M.J., Bingham, G., Gagou, M.E., Meuth, M., and Andrews, P.W. (2012). Human embryonic stem cells fail to activate CHK1 and commit to apoptosis in response to DNA replication stress. Stem Cells *30*, 1385–1393.

Desmarais, J.A., Unger, C., Damjanov, I., Meuth, M., and Andrews, P. (2016). Apoptosis and failure of checkpoint kinase 1 activation in human induced pluripotent stem cells under replication stress. Stem Cell Res. Ther. *7*, 17.

Draper, J.S., Smith, K., Gokhale, P., Moore, H.D., Maltby, E., Johnson, J., Meisner, L., Zwaka, T.P., Thomson, J.A., and Andrews, P.W. (2004). Recurrent gain of chromosomes 17q and 12 in cultured human embryonic stem cells. Nat. Biotechnol. *22*, 53–54.

Hinds, P.W., Mittnacht, S., Dulic, V., Arnold, A., Reed, S.I., and Weinberg, R.A. (1992). Regulation of retinoblastoma protein functions by ectopic expression of human cyclins. Cell *70*, 993–1006.

Ichijima, Y., Yoshioka, K., Yoshioka, Y., Shinohe, K., Fujimori, H., Unno, J., Takagi, M., Goto, H., Inagaki, M., Mizutani, S., and Teraoka, H. (2010). DNA lesions induced by replication stress trigger mitotic aberration and tetraploidy development. PLoS One *5*, e8821.

Janssen, A., Van der burg, M., Szuhai, K., Kops, G.J., and Medema, R.H. (2011). Chromosome segregation errors as a cause of DNA damage and structural chromosome aberrations. Science *333*, 1895–1898.

Lamm, N., Ben-David, U., Golan-Lev, T., Storchová, Z., Benvenisty, N., and Kerem, B. (2016). Genomic instability in human pluripotent stem cells arises from replicative stress and chromosome condensation defects. Cell Stem Cell *18*, 253–261.

Lundberg, A.S., and Weinberg, R.A. (1998). Functional inactivation of the retinoblastoma protein requires sequential modification by at least two distinct cyclin-cdk complexes. Mol. Cell Biol. *18*, 753–761.

Merkle, F.T., Ghosh, S., Kamitaki, N., Mitchell, J., Avior, Y., Mello, C., Kashin, S., Mekhoubad, S., Ilic, D., Charlton, M., et al. (2017). Human pluripotent stem cells recurrently acquire and expand dominant negative P53 mutations. Nature *545*, 229–233.

Olariu, V., Harrison, N.J., Coca, D., Gokhale, P.J., Baker, D., Billings, S., Kadirkamanathan, V., and Andrews, P.W. (2010). Modeling the evolution of culture-adapted human embryonic stem cells. Stem Cell Res. *4*, 50–56.

Pera, M.F., Reubinoff, B., and Trounson, A. (2000). Human embryonic stem cells. J. Cell Sci. *113*, 5.

Simara, P., Tesarova, L., Rehakova, D., Matula, P., Stejskal, S., Hampl, A., and Koutna, I. (2017). DNA double-strand breaks in human induced pluripotent stem cell reprogramming and long-term in vitro culturing. Stem Cell Res. Ther. *8*, 73.

Taapken, S.M., Nisler, B.S., Newton, M.A., Sampsell-Barron, T.L., Leonhard, K.A., Mcintire, E.M., and Montgomery, K.D. (2011). Karyotypic abnormalities in human induced pluripotent stem cells and embryonic stem cells. Nat. Biotechnol. *29*, 313–314.

Takano, Y., Kato, Y., Van Diest, P.J., Masuda, M., Mitomi, H., and Okayasu, I. (2000). Cyclin D2 overexpression and lack of p27 correlate positively and cyclin E inversely with a poor prognosis in gastric cancer cases. Am. J. Pathol. *156*, 585–594.

Thompson, O., Von Meyenn, F., Hewitt, Z., Alexander, J., Wood, A., Weightman, R., Gregory, S., Krueger, F., Andrews, S., Barbaric, I., and Gokhale, P.J. (2020). Low rates of mutation in clinical grade human pluripotent stem cells under different culture conditions. Nat. Commun. *11*, 1528.

Vallabhaneni, H., Lynch, P.J., Chen, G., Park, K., Liu, Y., Goehe, R., Mallon, B.S., Boehm, M., and Hursh, D.A. (2018). High basal levels of γH2AX in human induced pluripotent stem cells are linked to replication-associated DNA damage and repair. Stem Cells *36*, 1501–1513.

Zeman, M.K., and Cimprich, K.A. (2014). Causes and consequences of replication stress. Nat. Cell Biol. *16*, 2–9.

Zhang, J., Hirst, A.J., Duan, F., Qiu, H., Huang, R., Ji, Y., Bai, L., Zhang, F., Robinson, D., Jones, M., et al. (2019). Anti-apoptotic mutations desensitize human pluripotent stem cells to mitotic stress and enable aneuploid cell survival. Stem Cell Rep. *12*, 557–571.

Stem Cell Reports, Volume *14*

Supplemental Information

Nucleosides Rescue Replication-Mediated Genome Instability of Human Pluripotent Stem Cells

Jason A. Halliwell, Thomas J.R. Frith, Owen Laing, Christopher J. Price, Oliver J. Bower, Dylan Stavish, Paul J. Gokhale, Zoe Hewitt, Sherif F. El-Khamisy, Ivana Barbaric, and Peter W. Andrews

Supplemental Figures

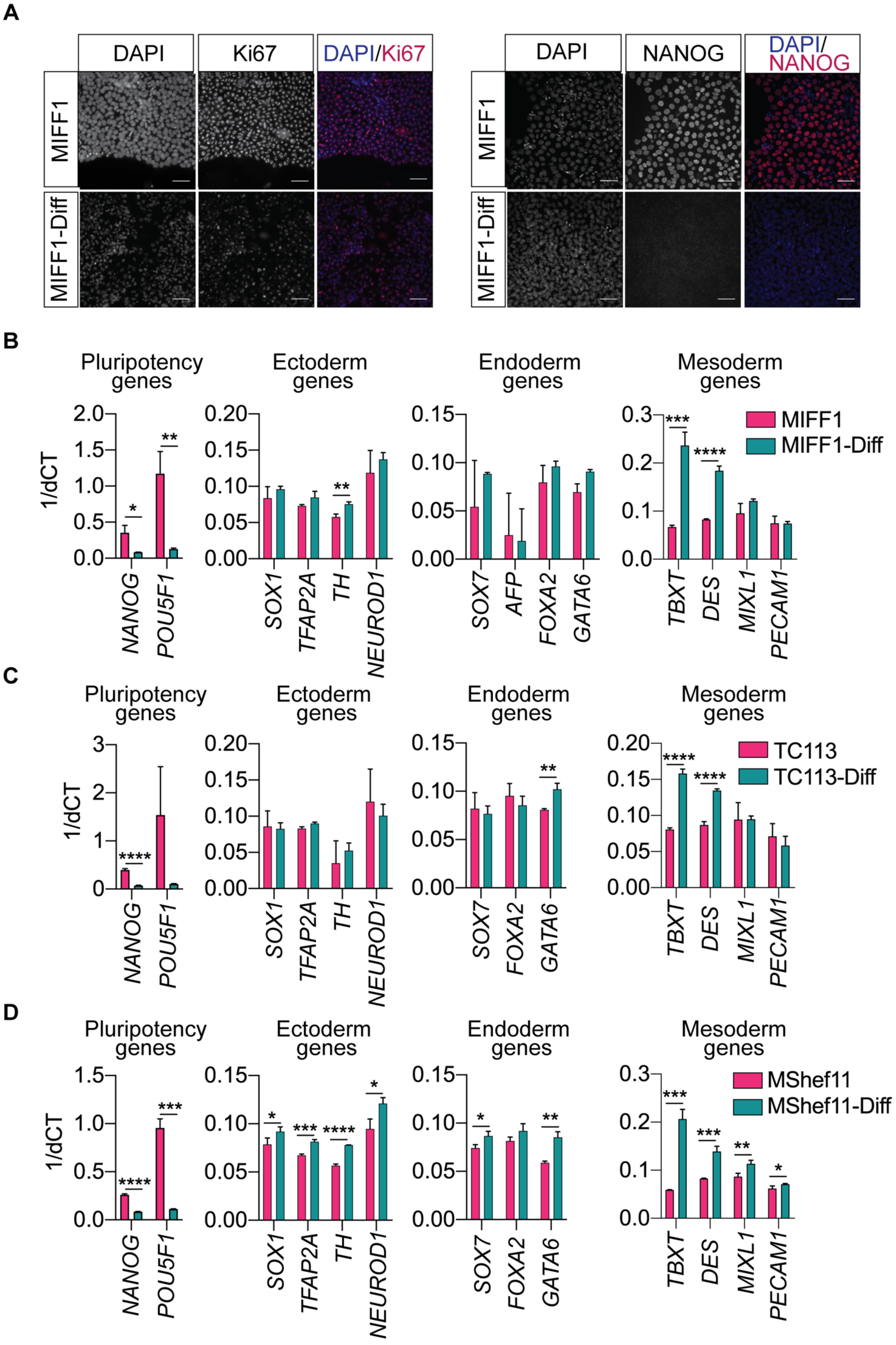

Figure S1| Differentiation of human PSC lines to obtain isogenic differentiated derivatives. Figure S1 supports figure 1 by providing details of the human PSC differentiation A, Representative immunofluorescence images of MIFF1 and MIFF1 Differentiated cells (MIFF1-Diff) stained for Ki67 and NANOG, with nuclei counterstained with Hoechst 33342. Scale bar, 50μm. **B-D,** RT-qPCR gene expression data of MIFF1 **(B),** TC113 **(C)** and MShef11 **(D)** compared to their differentiated derivatives. Genes associated with pluripotency, ectoderm, endoderm and mesoderm are displayed (left to right). Data in **B**-**D** are mean ± s.d., two-tailed *t*-test, *P<0.05, **P<0.01, ***P<0.001, ****P<0.0001, (*n* = 3 experiments).

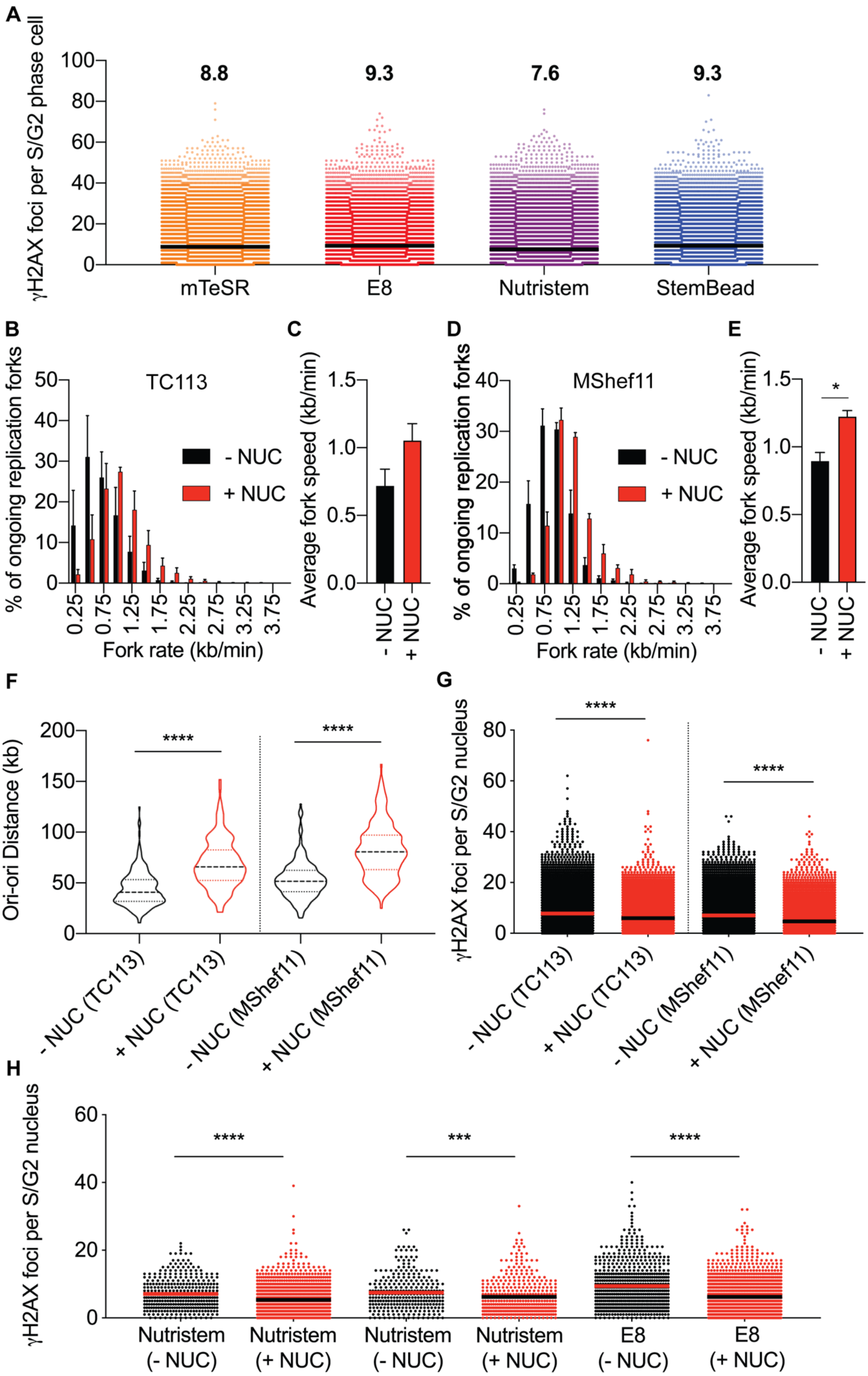

A
γH2AX foci per S/G2 phase cell
8.8
9.3
7.6
9.3
mTeSR
E8
Nutristem
StemBead
B
% of ongoing replication forks
TC113
- NUC
+ NUC
Fork rate (kb/min)
C
Average fork speed (kb/min)
- NUC
+ NUC
D
% of ongoing replication forks
MShef11
- NUC
+ NUC
Fork rate (kb/min)
E
Average fork speed (kb/min)
*
- NUC
+ NUC
F
Ori-ori Distance (kb)
****
****
- NUC (TC113)
+ NUC (TC113)
- NUC (MShef11)
+ NUC (MShef11)
G
γH2AX foci per S/G2 nucleus
****
****
- NUC (TC113)
+ NUC (TC113)
- NUC (MShef11)
+ NUC (MShef11)
H
γH2AX foci per S/G2 nucleus
****
***
****
Nutristem (- NUC)
Nutristem (+ NUC)
Nutristem (- NUC)
Nutristem (+ NUC)
E8 (- NUC)
E8 (+ NUC)

Figure S2 | Replication stress and a susceptibility to DNA damage is a characteristic of undifferentiated human PSC, Figure S2 additional data in support of figure 3. A, The number of γH2AX foci per S/G2 phase cell when MIFF1 was grown in mTeSR, E8, Nutristem or StemBeads. The S/G2 phase was determined from nuclear DNA content. Data points represent individual MIFF1 cells and are the results from three independent experiments, centre line indicates the mean, the mean numerical value is also presented above each plot. The mean numbers of γH2AX foci was similar for all media, except that it was slightly lower in the case of Nutristem, two-tailed *t*-test, ****P<0.0001. **B-F,** Additional data from DNA fibre assays performed on TC113 **(B,C)** and MShef11 **(D,E)** in absence of exogenous nucleosides (- NUC) or in the presence of exogenous nucleoside (+ NUC)**. B,D** Distribution of replication fork rates (*n* > 200 forks per cell line per experiment, *n* = 3 experiments), data presented is the mean value from each experiment ± s.e.m. **C,E** Mean fork rates. Data is mean ± s.d., two-tailed *t*-test, *P<0.05**.** (*n* > 200 forks per cell line per experiment, *n* = 3 experiments) **F,** Distribution of adjacent origins distance measurements (Ori-ori). Median distance, 25th and 75th quartiles are presented, two-tailed *t*-test, ****P<0.0001 (*n* > 160 per cell line, *n* = 3 experiments). **G,** Number of γH2AX foci per S/G2 phase cell (determined from DNA content). Comparison made between TC113 and MShef11 cells grown without nucleosides (- NUC) and with nucleoside (+ NUC). Data in **G** are the results from 3 independent experiments, centre line is the mean. two-tailed *t*-test, ****P<0.0001 (*n* > 100 cells per cell line per experiment). **H,** The number of γH2AX foci per S/G2 phase MIFF1 cell when grown in Nutristem or E8 without the addition of nucleosides (- NUC) and when these media were supplemented with exogenous nucleosides (+ NUC). Each plot shows an individual experiment, with each point representing the value from a single MIFF1 cell. Centre line indicates the mean. two-tailed *t*-test, ***P<0.001, ****P<0.0001 (*n* > 100 cells per experiment).

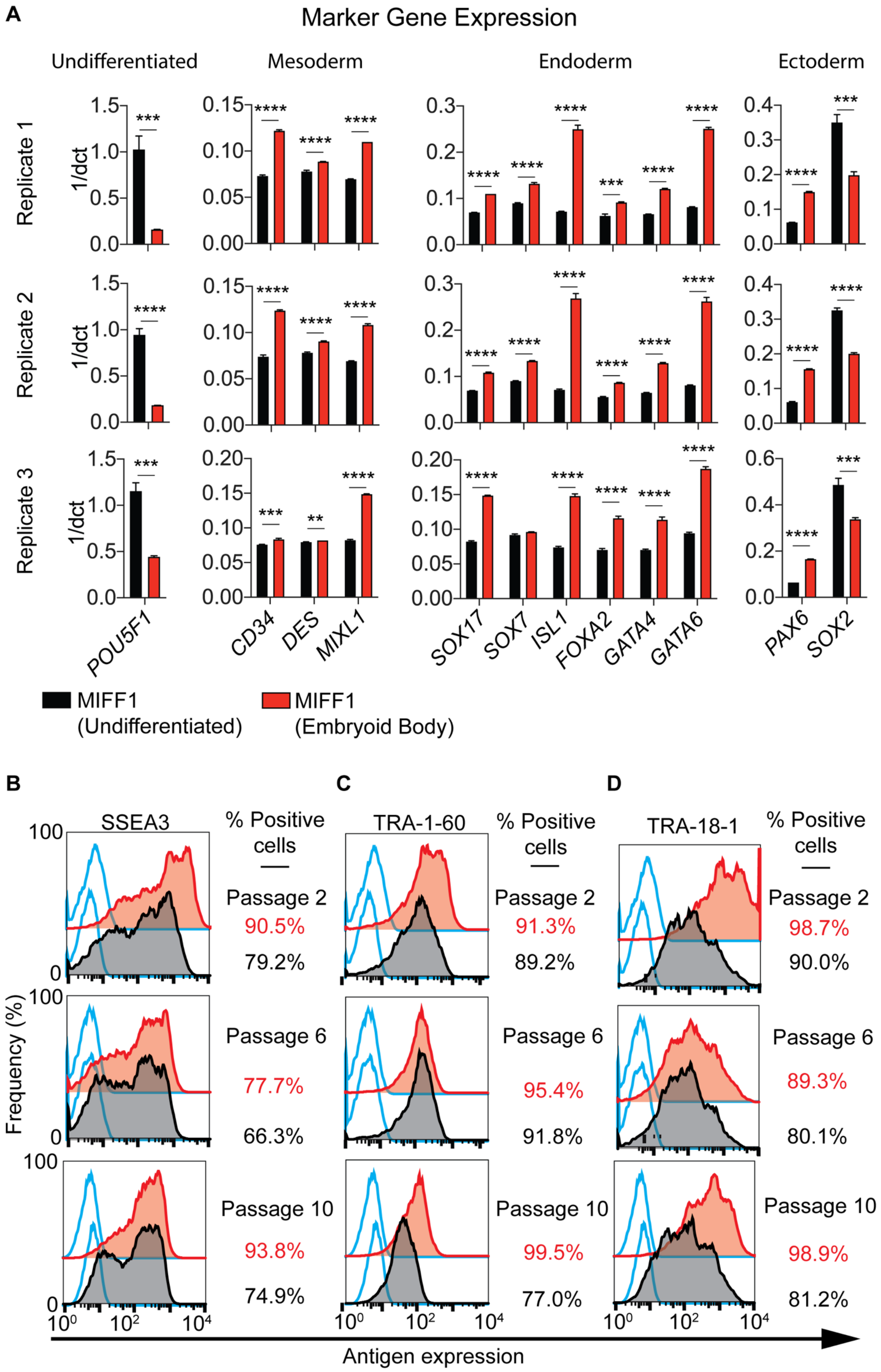

A
Marker Gene Expression
Undifferentiated
Mesoderm
Endoderm
Ectoderm
Replicate 1
Replicate 2
Replicate 3
1/dct
POU5F1
CD34
DES
MIXL1
SOX17
SOX7
ISL1
FOXA2
GATA4
GATA6
PAX6
SOX2
MIFF1 (Undifferentiated)
MIFF1 (Embryoid Body)
B
C
D
SSEA3
TRA-1-60
TRA-18-1
% Positive cells
Passage 2
90.5%
79.2%
91.3%
89.2%
98.7%
90.0%
Passage 6
77.7%
66.3%
95.4%
91.8%
89.3%
80.1%
Passage 10
93.8%
74.9%
99.5%
77.0%
98.9%
81.2%
Frequency (%)
Antigen expression

Figure S3 | Human PSC retain pluripotency associated antigen expression and the ability to differentiate into the three germ layers when cultured in exogenous nucleosides, additional data to support figure 3. A, Embryoid bodies were produced by differentiation under neutral conditions for 10 days from MIFF1 cells cultured with exogenous nucleosides for ten passages prior to differentiation. RT-qPCR analysis of marker gene expression by the embryoid bodies compared to gene expression in undifferentiated MIFF1 cells from which the EBs were derived. Genes associated with undifferentiated cells, mesoderm**,** endoderm and ectoderm are displayed. Each row shows the results from an individual biological replicate. Data of marker gene expression are means ± s.d., two-tailed *t*-test, *<P0.05, **P<0.01, ***P<0.001, ****P<0.0001 (*n* = 3 technical replicates per experiment). **B-D,** Pluripotency associated antigen expression, human PSC are grown with exogenous nucleosides (+ NUC) (Red) versus the same stem cell culture media in the absence of exogenous nucleosides (- NUC) (Black) over 10 passages. Baseline fluorescence was set using the primary antibody control, P3X (Blue). Percentage positive population is displayed to the right of each histogram. **B,** SSEA3 antigen expression from passage 2 to passage 10 (top to bottom). **C,** TRA-1-60 antigen expression passage 2 to 10. **D,** Tra-18-1 antigen expression from passage 2 to passage 10. **B-D,** Modal scaled channels, histograms are displayed as a percentage of the maximum count.

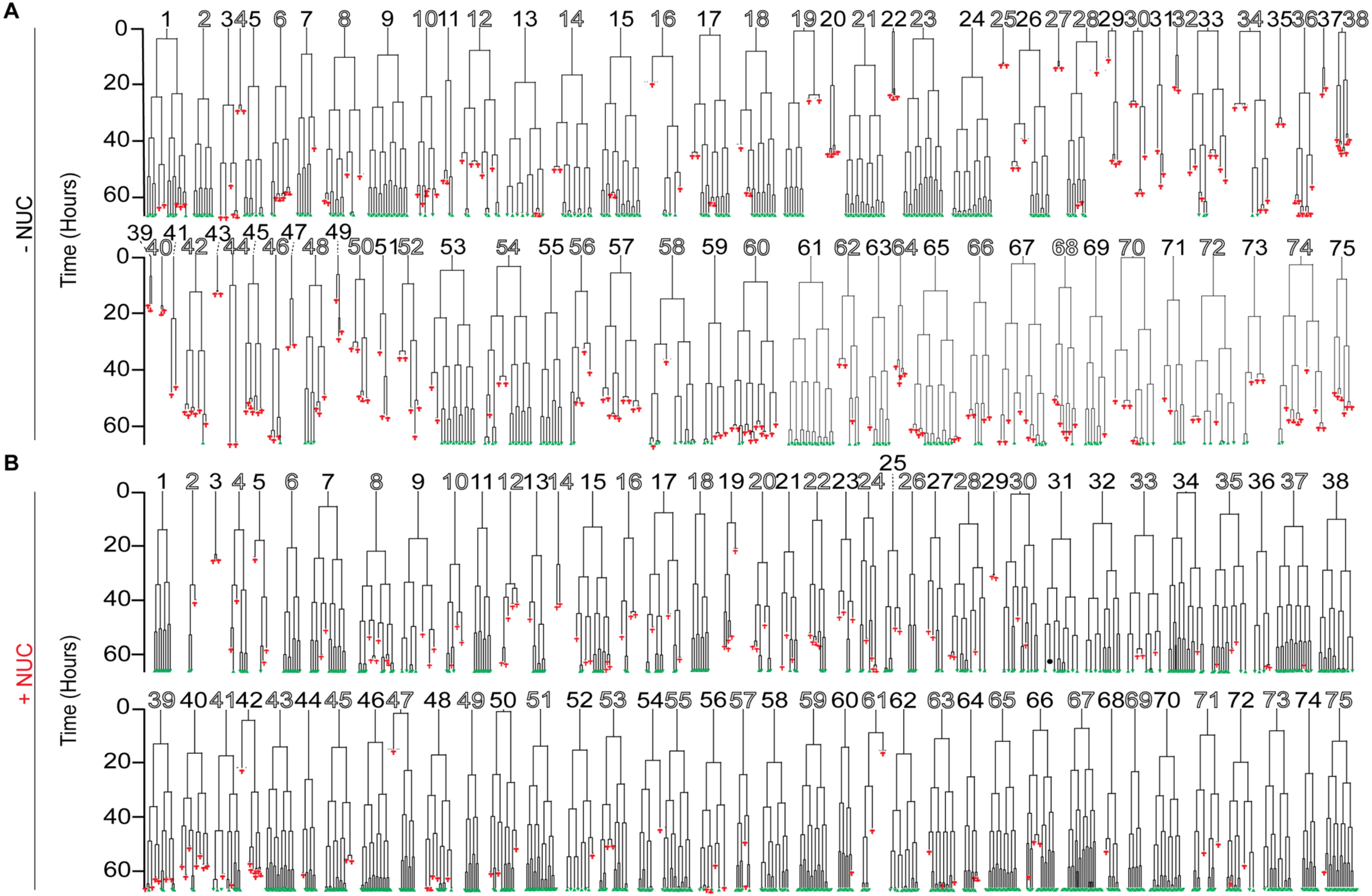
A
B
- NUC
+ NUC
Time (Hours)
Time (Hours)
0
20
40
60

Figure S4 | Exogenous nucleosides improve survival and plating efficiency of human PSC, figure S4 displays data from figure 4a in full. A,B, Lineage trees of the 75 cells, that reached the first cell division, sampled from MIFF1 grown in the absence of exogenous nucleosides (- NUC) (**A**) and when grown in the presence of exogenous nucleosides (+ NUC) (**B**) conditions. Twenty randomly selected starting cells and the resulting lineage trees are displayed in Figure 4.

Supplemental Experimental Procedures

Immunocytochemistry. Cells were fixed with 4% paraformaldehyde (Sigma, 158127) for 10 minutes. Cells were permeabilised and blocked with 0.3% Triton-X (Sigma, T8787), 10% goat serum (Thermo Fisher Scientific, 16210072) and 3% BSA (Sigma) in PBS for 1 hour. Primary antibody incubation was performed overnight at 4°C: Anti-Phospho-Histone H2A.X (Ser139) (Cell Signalling Technologies, 9718; 1:400), Anti-gamma H2A.X (Phospho S139) (abcam, ab26350; 1:500), Anti-Nanog (Cell Signalling, 4903; diluted 1:500), Anti-Nanog (Cell Signalling, 4893; diluted 1:500) and Anti-Ki67 (abcam, ab238020; diluted 1:100). Secondary antibody incubation was performed for 1 hour: Alexa Fluor 488-conjugated anti-rabbit IgG (Life Technologies, A11034; diluted 1:400), Alexa Fluor 647 AffiniPure Goat anti-Rabbit IgG (H+L) (Jackson Immuno Research, 111-605-003; 1:1000) and Alexa Fluor 647 AffiniPure Goat anti-Mouse IgG (H+L) (Jackson Immuno Research, 115-605-003; 1:400). All antibodies were diluted in 1% BSA (Sigma) and 0.3% Triton-X (Sigma, T8787) in PBS. Nuclei were counterstained with Hoechst 33342 (Thermo Fisher Scientific, H3570; diluted 1:1000) and images were acquired using an INCell Analyzer 2200 (GE Healthcare) high content microscope taking 25 or 30 randomized images per well.

Immunofluorescence data analysis. Where possible, CellProfiler(Carpenter et al., 2006) cell image analysis software was used to analyse the high content images. Immunofluorescence was quantified above a threshold set by a secondary only control. Hoechst 33342 was used to identify the cells nuclei and mask the nuclear area over the immunofluorescence staining. Where it was necessary to separate cells by cell cycle stage the integrated intensity of the stained nuclei was calculated using CellProfiler Analyst (Jones et al., 2008).

Neutral comet assay. 150μL of 0.6% agarose (Sigma, A9539) was set on a fully frosted glass slide, sandwiched beneath a coverslip. Once dried, 12,000 cells per conditions was resuspended in 75μL ice cold PBS and mixed with 75μL of 1.2% low melting agarose (Sigma, A4018). The cell and agarose suspension was mounted on top of the original agarose layer beneath a coverslip and set at 4°C. The slides were immersed in pre-chilled lysis buffer (2.5M NaCl, 10mM Tris-HCL, 100mM EDTA pH8.0, 0.5% Triton-X, 3% DMSO) for 1.5 hours at 4°C, washed in H_2O and equilibrized in electrophoresis buffer (300mM sodium acetate, 100mM Tris-EDTA and 1% DMSO) for 1 hour. Electrophoresis is performed at 25V for 1 hour. Slides were stained with SYBR green (Sigma, S9430), imaged and quantified using Comet Assay IV (Instem) live video measurement system.

DNA fibre assay. DNA fibre assay was performed as previously described (Groth et al., 2010). Briefly, cells were plated and grown for a minimum of 72 hours before sequential pulse labelling with 2.5mM CldU (Sigma, C6891; 1:100) and then 2.5mM IdU (Sigma, I7125; 1:10) for 20 minutes each. Cells were washed with ice cold PBS, dissociated using TrypLE cell dissociation enzyme (Thermo Fisher Scientific, 12504013) and diluted to $4x10^5$ cells/ml in cold PBS. To spread the labelled fibers, 2μL of cell suspension was dropped onto a glass slide and allowed to dry for 5-7 minutes before adding 7μL of spreading buffer (200mM Tris-HCL PH7.4, 50mM EDTA, 0.5% SDS). The solutions were mixed with a pipette tip and incubated for 2 minutes. Slides were tilted at an angle of 25° and the droplet timed to ensure consistent spreading was achieved. Slides were air dried and fixed with 3:1 methanol/acetic acid. For the immunostaining, the glass slides were first washed twice with H_2O for 5 minutes each, denatured with 2.5M HCL for 1 hour and then blocked in 1% BSA (Sigma) and 0.1% Tween20. Primary antibodies were incubated for 1 hour: Rat anti-BrdU, clone BU1/75 (Novus Biologicals NB500-169) (AbD Serotec; diluted 1:400) or Anti-BrdU clone BU1/75 (ICR1) (abcam, ab6326; diluted 1:400) and Mouse anti-BrDU (Clone B44) (Becton Dickinson, 347580; diluted 1:250). The secondary antibodies used were Alexa Fluor 555 goat anti-rat IgG (Thermo Fisher Scientific, A21434; diluted 1:500) and Alexa Fluor 488 F (ab')2-Goat anti-Mouse IgG (Thermo Fisher Scientific, A-11017; diluted 1:500). Slides were mounted with Fluoroshield (Sigma, F6182), and images were acquired using Olympus FV1000 confocal microscope.

RNA Extraction and reverse transcriptase qPCR. RNA was extracted using Qiagen RNeasy kit. cDNA synthesis was performed using high capacity reverse transcription kit (Thermo Fisher Scientific, 4368814). qPCR was performed in 384 well plates with 10μL reactions consisting of 1X TaqMan Fast Universal Master Mix (ThermoFisher, 4352042), 100nM of forward and reverse primers (Table S1), 100nM of probe from the Universal Probe Library (Roche) and 2μL of 5ng/μL cDNA. PCR reactions were run on the QuantStudio 12K Flex Thermocycler (Life Technologies 4471087). All reactions were performed in triplicate with comparative Ct normalized to GAPDH or B-ACTIN expression.

Table S1. Primer sequences and Universal probe library probes used in this study that relate to Figure 2, S2 and S6.

Gene	Sense	Anti-sense	Probe
POU5F1	agcaaaacccggaggagt	ccacatcggcctgtgtatatc	35
NANOG	agatgcctcacacggagact	tttgcgacactcttctctgc	31
SOX17	cgccgagttgagcaagat	ggtggtcctgcatgtgct	13
TFAP2A	acatgctcctggctacaaaac	aggggagatcggtcctga	62
TH	tcagtgacgccaaggaca	gtacgggtcgaacttcacg	42
NEUROD1	acctcgaagccatgaacg	cttccaggtcctcatcttcg	55
SOX7	ttcctcaccagccaggtc	atttgcgggaagttgctcta	30
AFP	tgtactgcagagataagtttagctgac	tccttgtaagtggcttcttgaac	61
FOXA2	cgccctactcgtacatctcg	agcgtcagcatcttgttgg	9
GATA6	aatacttcccccacaacacaa	ctctcccgcaccagtcat	90
MIXL1	gacacagatgaggggcagtt	cccgttttcagctaccattc	6
BRACHYURY	aggtacccaaccctgagga	gcaggtgagttgtcagaataggt	23
DES	ggagattgccacctaccg	ggtctggatggggagattg	55
PECAM1	ggtctggatggggagattg	ttcaagtttcagaatatcccaatg	37
GAPDH	agccacatcgctcagacac	gcccaatacgaccaaatcc	60
B-ACTIN	ccaaccgcgagaagatga	ccagaggcgtacagggatag	64
CCND2	agctgctggctaagatcacc	acggtctgctgcaggctat	68
CCNE1	ggccaaaatcgacaggac	catcatcttctttgtcaggtgtg	32

Western blotting. Laemili buffer (4% SDS, 20% Glycerol, 0.125M Tris HCl, 0.004% bromphenol blue) was added to cell pellets and sonicated for 10 seconds. Protein lysate was incubated for 10 minutes at 95°C and concentration was determined by NanoDrop spectrophotometer (Thermo Fisher Scientific).
Protein was separated on 10% ProtoGel (National Diagnostics) run at 120V for 1.5 hours and transferred onto PVDF membrane (Millipore, #IPVH00010). Primary antibodies were incubated over night at 4°C: α-Tubulin (Cell Signalling Technology, 2144; diluted 1:1000), Cyclin E1 (D7T3U) (Cell Signalling Technology, 20808; diluted 1:500), Cyclin D2 (D52F9) (Cell Signalling Technology, 3741; diluted 1:500). The blot was washed and incubated with Anti-rabbit IgG or anti-mouse IgG secondary antibody for 1 hour (Promega, W401 & W402). Immunoreactivity was visualised with ECL prime (GE Healthcare, RPN2232) on a CCD-based camera.

The generation of hPSC stably expressing H2B-RFP. Transfection with pCAG-H2B-RFP-IRES-PURO vector (Liew et al., 2007) was performed using electroporation. Cells were electroporated with a single 1600V pulse for 20msec using the Neon transfection system according to manufacturer's instructions (Thermo Fisher Scientific, MPK10025). Stable clones were obtained by puromycin selection. Puromycin concentration was increased gradually over 5 days to a final concentration of 0.375μg/mL before flow sorting for the brightest population of RFP.

Supplemental References

CARPENTER, A. E., JONES, T. R., LAMPRECHT, M. R., CLARKE, C., KANG, I. H., FRIMAN, O., GUERTIN, D. A., CHANG, J. H., LINDQUIST, R. A., MOFFAT, J., GOLLAND, P. & SABATINI, D. M. 2006. CellProfiler: image analysis software for identifying and quantifying cell phenotypes. *Genome Biol,* 7**,** R100.

GROTH, P., AUSLÄNDER, S., MAJUMDER, M. M., SCHULTZ, N., JOHANSSON, F., PETERMANN, E. & HELLEDAY, T. 2010. Methylated DNA causes a physical block to replication forks independently of damage signalling, O(6)-methylguanine or DNA single-strand breaks and results in DNA damage. *J Mol Biol,* 402**,** 70-82.

JONES, T. R., KANG, I. H., WHEELER, D. B., LINDQUIST, R. A., PAPALLO, A., SABATINI, D. M., GOLLAND, P. & CARPENTER, A. E. 2008. CellProfiler Analyst: data exploration and analysis software for complex image-based screens. *BMC Bioinformatics,* 9**,** 482.

LIEW, C. G., DRAPER, J. S., WALSH, J., MOORE, H. & ANDREWS, P. W. 2007. Transient and stable transgene expression in human embryonic stem cells. *Stem Cells,* 25**,** 1521-8.
